# Exact Closed-Form Multitarget Bayes Filters

**DOI:** 10.3390/s19122818

**Published:** 2019-06-24

**Authors:** Ronald Mahler

**Affiliations:** Random Sets LLC, Eagan, MN 55122, USA; MahlerRonald@comcast.net

**Keywords:** multitarget tracking, random finite set, finite-set statistics, undetected targets

## Abstract

The finite-set statistics (FISST) foundational approach to multitarget tracking and information fusion has inspired work by dozens of research groups in at least 20 nations; and FISST publications have been cited tens of thousands of times. This review paper addresses a recent and cutting-edge aspect of this research: exact closed-form—and, therefore, provably Bayes-optimal—approximations of the multitarget Bayes filter. The five proposed such filters—generalized labeled multi-Bernoulli (GLMB), labeled multi-Bernoulli mixture (LMBM), and three Poisson multi-Bernoulli mixture (PMBM) filter variants—are assessed in depth. This assessment includes a theoretically rigorous, but intuitive, statistical theory of “undetected targets”, and concrete formulas for the posterior undetected-target densities for the “standard” multitarget measurement model.

## 1. Introduction

Suppose that, at times *t*_1_, …, *t_k_*, a single sensor collects a time-series **z**_1:*k*_: **z**_1_, …, **z***_k_* of measurements from a target with dynamically evolving state **x**. Then, the Bayes-optimal approach for tracking the target is the recursive Bayes filter:

… →*f_k−_*_1*|k−*1_(**x**|**z**_1:*k−*1_) →*f_k|k_*_−1_(**x**|**z**_1:*k*−1_) →*f_k|k_*(**x**|**z**_1:*k*_) → …

where *f_k|k_*(**x**|**z**_1*:k*_) is the probability distribution of the unknown state **x** at time *t_k_*; where:

(1) $$f_{k|k-1}(x|z_{1:k-1})=\int f_{k|k-1}(x|x^{\prime })\cdot f_{k-1|k-1}(x^{\prime }|z_{1:k-1})dx^{\prime },\;f_{k|k}(x|z_{1:k})\propto f_{k}(z_{k}|x)\cdot f_{k|k-1}(x|z_{1:k-1})$$

and where *f_k|k−_*_1_(**x**|**x′**) is the target’s Markov state-transition density and *f_k_*(**z**|**x**) is the sensor’s measurement density. Suppose that these are linear-Gaussian:

(2) $$f_{k|k-1}(x|x^{\prime })=N_{R_{k}}(x-F_{k}x^{\prime }),\;f_{k}(z|x)=N_{Q_{k}}(z-H_{k}x).$$

Then the family of linear-Gaussian distributions solves the Bayes filter in exact closed form. That is, if the initial distribution is linear-Gaussian—i.e., if $f_{0|0}(x)=N_{P_{0|0}}(x-x_{0|0})$—then [1]:

(3) $$f_{k|k-1}(x|z_{1:k-1})=N_{P_{k|k-1}}(x-x_{k|k-1}),\;f_{k|k}(x|z_{1:k})=N_{P_{k|k}}(x-x_{k|k})$$

where …→ (**x***_k_*_−1|*k*−1_,*P_k_*_−1|*k*−1_) → (**x***_k_*_|*k*−1_,*P_k_*_|*k*−1_) → (**x***_k_*_|*k*_,*P_k_*_|*k*_) →… is the Kalman filter. The family of Gaussian mixture distributions also solves the Bayes filter in exact closed form [2].

An unexpected recent development has been the generalization of this approach to the multitarget case. Let:

… →*f_k−_*_1*|k−*1_(*X*|*Z*_1:*k−*1_) →*f_k|k−_*_1_(*X*|*Z*_1:*k−*1_) →*f_k|k_*(*X*|*Z*_1:*k*_) →…

be the multitarget recursive Bayes filter, where *f_k|k_*(*X*|*Z*_1:*k*_) is the probability distribution of the unknown multitarget state-set *X* and *Z*_1:*k*_:*Z*_1_, …, *Z_k_* is the time-sequence of collected multitarget measurement-sets. Suppose that *F* is a family of parametrized multitarget distributions *f*(*X*|**p**) with parameter **p** ∈ ℘. Then *F* solves the multitarget Bayes filter in exact closed form if *f_k|k_*_−1_(*X*|*Z*_1:*k*__−1_) = *f*(*X*|**p***_k_*_|*k*−1_) and *f_k|k_*(*X*|*Z*_1:*k*_) = *f*(*X*|**p***_k_*_|*k*_) with **p***_k_*_|*k*-1_,**p***_k_*_|*k*_∈ ℘. In this case, the multitarget Bayes filter can be replaced by an equivalent, but potentially computationally more tractable, filter: …→**p***_k−_*_1|*k*-1_→**p***_k_*_|*k*−1_→**p***_k_*_|*k*_→….

**Remark** **1.** *References [3,4,5,6,7,8,9] employ the terminology “conjugate filter” rather than “exact closed-form filter.” The latter usage is more accurate since “conjugate” refers specifically to exact algebraic closure of F with respect to f_k_(Z_k_|X) (and not f_k|k_* *_−1_(X|X* *′)).*

Five such filters have been proposed (where the earliest-published papers are indicated):

1. Generalized labeled multi-Bernoulli (GLMB) filter [8].
2. Labeled multi-Bernoulli mixture (LMBM) filter [10].
3. Poisson multi-Bernoulli mixture (PMBM) filter, in three distinct versions:
  - “Unlabeled” or U-PMBM filter [11].
  - “Label-augmented” or LA-PMBM filter [12].
  - “Hybrid labeled-unlabeled” or H-PMBM filter [4].

The purpose of this review paper is to provide an in-depth assessment of these five filters, especially in regard to the following questions: Is this filter theoretically rigorous? Is it a true multitarget tracker? Is it actually exact closed-form?

The basic issue distinguishing (3a, 3b, 3c) from (1, 2) is the form of the initial multitarget distribution and the target-birth model: Poisson or non-Poisson? In particular, the Poisson component of the PMBM distribution *f_k|k_*(*X*|*Z*_1:*k−*1_) is claimed to be a model of the “undetected targets” at time *t_k_*—i.e., those targets never detected at times *t*_1_, …, *t_k_*.

This physical interpretation forces us to address the following question: What is an “undetected target”? This, in turn, requires the formal statistical theory of “undetected targets” developed in Section 5. This theory results in the following formulas for the probability generating functionals (PGFLs) of the measurement-updated random finite set (RFS) Ξ*_k|k_* and its associated detected-target RFS $\Xi_{k|k}^{d}$ and undetected-target RFS $\Xi_{k|k}^{u}$(Section 5.7):

(4) $$G_{k|k}[h|Z_{1:k}]\propto \int f_{k}^{*}(Z_{k}|X)\cdot {\left(p_{D}h\right)}^{X}\cdot \frac{\delta G_{k|k-1}}{\delta X}[h(1-p_{D})]\delta X$$

(5) $$G_{k|k}^{d}[h|Z_{1:k}]\propto \int f_{k}^{*}(Z_{k}|X)\cdot {\left(p_{D}h\right)}^{X}\cdot \frac{\delta G_{k|k-1}}{\delta X}[1-p_{D}]\delta X$$

(6) $$G_{k|k}^{u}[h|Z_{1:k}]\propto \int f_{k}^{*}(Z_{k}|X)\cdot p_{D}^{X}\cdot \frac{\delta G_{k|k-1}}{\delta X}[h(1-p_{D})]\delta X.$$

The major conclusions of the paper are as follows:

1. The GLMB, LMBM filters solve the labeled multitarget Bayes filter in exact closed form.
2. They are, therefore, true multitarget trackers.
3. The U-PMBM filter solves the unlabeled multitarget Bayes filter in exact closed form.
4. The “undetected-targets” interpretation of the U-PMBM filter appears to be valid.
5. It is theoretically impossible to prune U-PMBM distributions in a practical manner.
6. The U-PMBM, LA-PMBM and H-PMBM filters are not true multitarget trackers.
7. The LA-PMBM and H-PMBM filters are theoretically and physically questionable.
8. In particular, the H-PMBM filter does not solve the “hybrid” multitarget Bayes filter in exact closed form.

The paper is organized as follows: overview of FISST-based multitarget tracking (Section 2); the GLMB and LMBM filters (Section 3); the three versions of the PMBM filter (Section 4); a theory of undetected targets (Section 5); mathematical derivations (Section 6); and conclusions (Section 7).

## 2. Overview of FISST-Based Multitarget Tracking

This section summarizes those concepts necessary to understand the paper. Greater detail can be found in books [13,14,15,16], tutorials [17,18,19,20], and a short survey of advances ca. 2015 [21]. Additionally, systematic investigations of FISST vs. “point processes” can be found in [19,22] and of FISST vs. measurement-to-track approaches in [23,24]. 

The section is organized as follows: Random finite sets (Section 2.1); multitarget calculus (Section 2.2); important RFSs (Section 2.3); the multitarget Bayes filter (Section 2.4); and the PGFL form of the multitarget Bayes filter (Section 2.5).

### 2.1. Random Finite Sets (RFSs)

Let ℑ be a single-target state-space (e.g., a region of a Euclidean space) with **x**, **x**′ ∈ ℑ and let ℵ be the sensor measurement-space with **z** ∈ ℵ. Then the state of a multitarget system is represented as a finite subset *X* = {**x**_1_, …, **x***_n_*} ⊆ ℑ with *X* = ∅ for *n* = 0. The number of elements in *X* is denoted as |*X*|. In a Bayesian approach, unknown states are random variables. Thus, an unknown multitarget state is a random finite set (RFS) Ξ ⊆ ℑ. 

Similarly, the “measurement” collected from the targets in *X* is a finite subset *Z* = {**z**_1_, …, **z***_m_*} ⊆ ℵ with *Z =* ∅ for *m* = 0. Since measurement-sets are random, multitarget measurements will be represented as random finite measurement-sets Σ ⊆ ℵ.

**Remark** **2.** *It is sometimes claimed that multitarget states can be rigorously modeled as variable-length concatenated vectors* (**x**_1_, …, **x***_n_*) ∈ ∪ *_n_*_≥ 0_ ℑ*^n^. This is not the case—see Section 2.4 of [19].*

### 2.2. Multitarget Calculus

A *multitarget density function* is a function *f*(*X*) ≥ 0 of the finite-set variable *X* ⊆ ℑ such that the units of measurement of *f*(*X*) are *ι* ^−|*X*|^ where *ι* is the unit of measurement of ℑ. The *set integral* of *f*(*X*) is:

(7) $$\int f(X)\delta X=f(\emptyset )+\sum_{n\geq 1}\frac{1}{n!}\int f_{n}(x_{1},\ldots ,x_{n})dx_{1}\cdots dx_{n}$$

where *f_n_*(**x**_1_, …, **x***_n_*) = *f*({**x**_1_, …, **x***_n_*})/*n*! for distinct **x**_1_, …, **x***_n_* [14] (p. 361). Every random finite state-set Ξ has a multitarget probability distribution $f_{\Xi }$ (*X*): ∫$f_{\Xi }$ (*X)δX* = 1. The *cardinality distribution* of Ξ is:

(8) $$p_{\Xi }(n)=\mathrm{Pr}(|\Xi |=n)=\int_{|X|=n}f_{\Xi }(X)\delta X=\frac{1}{n!}\int f_{\Xi }(\{x_{1},\ldots ,x_{n}\})dx_{1}\cdots dx_{n}.$$

The *probability generating functional* (PGFL) of Ξ is, for “test functions” 0 ≤ *h*(**x**) ≤ 1:

(9) $$G_{\Xi }[h]=\int h^{X}\cdot f_{\Xi }(X)\delta X$$

where *h^X^* = 1 if *X* = ∅ and *h^X^* = Π**_x_**_∈__*X*_*h*(**x**) otherwise. The simplest nontrivial PGFLs are:

(10) s[h]=∫h⋅s(x)dx

where *s*(**x**) ≥ 0 is a density function on ℑ. The power functional *h^X^* satisfies the generalized binomial theorem [13] (Equation (3.6)):

(11) $${\left(h_{1}+h_{2}\right)}^{X}=\sum_{Y\subseteq X}h_{1}^{Y}\cdot h_{2}^{X-Y}.$$

The intuitive definition of the *functional derivative* of $G_{\Xi }$ [*h*] is:

(12) $$\frac{\delta G_{\Xi }}{\delta x}[h]=\underset{\varepsilon ↓0}{\lim}\frac{G_{\Xi }[h+\varepsilon \cdot \delta_{x}]-G_{\Xi }[h]}{\varepsilon }$$

where *δ***_x_**(**y**) is the Dirac delta function concentrated at **x**. (For a rigorous definition see [13,18].) If *X* = {**x**_1_, …, **x***_n_*} with |*X*|=*n* then the *general functional derivative* is $G_{\Xi }$ [*h*] if *X =* ∅ and, otherwise:

(13) $$\frac{\delta G_{\Xi }}{\delta X}[h]=\frac{\delta^{n}G_{\Xi }}{\delta x_{1}\cdots \delta x_{n}}[h]=\frac{\delta }{\delta x_{n}}\frac{\delta^{n-1}G_{\Xi }}{\delta x_{1}\cdots \delta x_{n-1}}[h].$$

The PGFL and multitarget distribution of an RFS are related by:

(14) $$f_{\Xi }(X)=\frac{\delta G_{\Xi }}{\delta X}[0].$$

The probability hypothesis density (PHD) of Ξ is:

(15) $$D_{\Xi }(x)=\int f_{\Xi }(\{x\}\cup X)\delta X=\frac{\delta G_{\Xi }}{\delta x}[1].$$

FISST includes an extensive “toolbox” of “turn-the-crank” rules for set integrals and functional derivatives—see [14] (pp. 383–389) or [13] (pp. 69–80).

### 2.3. Important RFSs

Various RFSs of importance to this paper are most easily described using their PGFLs:

1. Poisson RFS: $G_{\Xi }$ [*h*] = *e^D^*^[*h−*1]^ where *D*[*h*] = ∫*h*(**x**)⋅*D*(**x**)*d***x** and where *D*(**x**) ≥ 0 is a PHD—i.e., a density function on **x**∈ ℑ.
2. *Bernoulli RFS*: $G_{\Xi }$ [*h*] = 1 – *q* + *q*⋅*s*[*h*] where 0 ≤ *q* ≤ 1 and probability density *s*(**x**) are, respectively, the existence probability and spatial distribution of a single target.
3. *Multi-Bernoulli* (MB) *RFS*: $G_{\Xi }[h]=\prod_{i=1}^{N}\left(1-q_{i}+q_{i}\cdot s_{i}[h]\right)$ where 0 ≤ *q_i_* ≤ 1 and probability density *s_i_*(**x**) are, respectively, the existence probability and spatial distribution of the *i*-th of *N* targets.
4. *Multi-Bernoulli Mixture* (MBM) *RFS*: $G_{\Xi }[h]=\sum_{l=1}^{N}w_{l}\prod_{i=1}^{N_{l}}\left(1-q_{l.i}+q_{l,i}\cdot s_{l,i}[h]\right)$.
5. *Poisson Multi-Bernoulli* (PMB) *RFS*: $G_{\Xi }[h]=e^{D[h-1]}\prod_{i=1}^{N}\left(1-q_{i}+q_{i}\cdot s_{i}[h]\right)$.
6. *Poisson Multi-Bernoulli Mixture* (PMBM) *RFS*: $G_{\Xi }[h]=e^{D[h-1]}\sum_{l=1}^{N}w_{l}\prod_{i=1}^{N_{l}}\left(1-q_{l.i}+q_{l,i}\cdot s_{l,i}[h]\right)$.

### 2.4. Multitarget Recursive Bayes Filter

As noted earlier, this is:

… →*f_k−_*_1*|k−*1_(*X*|*Z*_1:*k−*1_) →*f_k|k−_*_1_(*X*|*Z*_1:*k−*1_) →*f_k|k_*(*X*|*Z*_1:*k*_) → …

where:

(16) $$f_{k|k-1}(X|Z_{1:k-1})=\int f_{k|k-1}(X|X^{\prime },Z_{1:k-1})\cdot f_{k-1|k-1}(X^{\prime }|Z_{1:k-1})\delta X^{\prime }$$

(17) $$f_{k|k}(X|Z_{1:k})\propto f_{k}(Z_{k}|X,Z_{1:k-1})\cdot f_{k|k-1}(X|Z_{1:k-1})$$

and where *f_k|k−_*_1_(*X*|*X*′,*Z*_1:*k*−1_) is the multitarget Markov state-transition density and *f_k_*(*Z*|*X*,*Z*_1:*k*−1_) is the sensor’s multitarget measurement density. It is usually assumed that *f_k|k−_*_1_(*X*|*X*′,*Z*_1:*k*−1_) = *f_k|k−_*_1_(*X*|*X*′) and *f_k_*(*Z*|*X*,*Z*_1:*k*−1_) = *f_k_*(*Z*|*X*); but the original forms allow (for example) the target-birth process and the clutter process, respectively, to be estimated from the measurements in *Z*_1:*k*−1_.

In this paper we will be concerned with *f_k_*(*Z*|*X*) for only the “standard” multitarget measurement model, which has PGFL:

(18) $$G_{k}[g|X]=\int g^{Z}\cdot f_{k}(Z|X)\delta Z=e^{\kappa_{k}[g-1]}\cdot {\left(1-p_{D}^{k}+p_{D}^{k}L_{g}^{k}\right)}^{X}.$$

Here, at time *t_k_*, $p_{D}^{k}(x)$ is the sensor probability of detection, $f_{k}(z|x)=L_{z}^{k}(x)$ is the sensor measurement density, *k_k_*(**z**) is the intensity function of a Poisson clutter process, and $L_{g}^{k}(x)=\int g(z)\cdot f_{k}(z|x)dz$. For notational simplicity we will usually suppress the time-index *k*—e.g., $p_{D}^{k}(x)=p_{D}(x),$$L_{z}^{k}(x)=L_{z}(x),$ etc.

Likewise, we will be concerned with *f_k|k−_*_1_(*X*|*X*′) for only the “standard” multitarget motion model, which has PGFL:

(19) $$G_{k|k-1}[h|X^{\prime }]=\int h^{X}\cdot f_{k|k-1}(X|X^{\prime })\delta X=G_{B}^{k|k-1}[h]\cdot {\left(1-p_{S}^{k|k-1}+p_{S}^{k|k-1}M_{h}^{k|k-1}\right)}^{X^{\prime }}.$$

Here, at time *t_k_*, $p_{S}^{k|k-1}(x^{\prime })$ is the target probability of survival, $f_{k|k-1}(x|x^{\prime })=M_{x}^{k|k-1}(x^{\prime })$ is the target Markov density, $G_{B}^{k|k-1}[h]$ is the PGFL of a multitarget birth RFS, and $M_{h}^{k|k-1}(x^{\prime })=\int h(x)\cdot f_{k|k-1}(x|x^{\prime })dx$. For notational simplicity we will usually suppress the time-index *k*—e.g., $p_{S}^{k|k-1}=p_{S}$, $M_{x}^{k|k-1}=M_{x}$, etc.

### 2.5. PGFL Form of the Multitarget Bayes Recursive Filter

The PGFL form of Equation (16) for the standard motion model is [14] (Equation (14.273)), [13] (Equation (5.94)):

(20) $$G_{k|k-1}[h|Z_{1:k-1}]=G_{B}^{k|k-1}[h]\cdot G_{k-1|k-1}[1-p_{S}+p_{S}M_{h}|Z_{1:k-1}].$$

The PGFL form of Equation (17) is [14] (Equation (14.280)), [13] (Equation (5.58)):

(21) $$G_{k|k}[h|Z_{1:k}]=\frac{\frac{\delta F_{k}}{\delta Z_{k}}[0,h]}{\frac{\delta F_{k}}{\delta Z_{k}}[0,1]}=\frac{{\left[\frac{\delta F_{k}}{\delta Z_{k}}[g,h]\right]}_{g=0}}{{\left[\frac{\delta F_{k}}{\delta Z_{k}}[g,h]\right]}_{g=0.h=1}}$$

where, for the standard measurement model [14] (Equation (14.290)), [13] (Equation (5.104)):

(22) $$F_{k}[g,h]=e^{\kappa_{k}[g-1]}\cdot G_{k-1|k}[h(1-p_{D}+p_{D}L_{g})|Z_{1:k-1}].$$

In what follows, we will notationally suppress the dependence of these PGFLs on *Z*_1:*k*−1_.

## 3. The GLMB and LMBM Filters

The section is organized as follows: labeled RFSs (Section 3.1); important labeled RFSs (Section 3.2); the GLMB filter (Section 3.3); and the LMBM filter (Section 3.4).

### 3.1. Labeled Random Finite Sets (LRFSs)

*Track labeling* (or, more generally, target identity) in an RFS context was first addressed in 1997 in [25] (pp. 135, 196–197) and in 2007 in [14] (pp. 505–508). However, the first implementations of RFS filters did not take track labels into account because of computational concerns. Later implementations, such as the Gaussian mixture cardinalized probability hypothesis density (GM-CPHD) filter, addressed labeling heuristically [13] (pp. 244–250). The labeling issue was not addressed in a theoretically rigorous and systematic fashion until 2011 in the *labeled RFS* (LRFS) papers of Vo and Vo [7,8].

In LRFS theory, single-target states are assumed to have the form **x** = (**u**,ℓ) ∈ ℑ × *L*_0_ where **u** ∈ ℑ is a kinematic target state-vector and ℓ is an element of a countable set *L*_0_ of target labels. The integral on ℑ × *L*_0_ is defined by:

(23) $$\int f(x)dx=\sum_{\ell \in L_{\infty }}f(u,\ell )du$$

where, by assumption, ∫*f*(**u**,ℓ)*d***u** = 0 for all but a finite number of ℓ. The corresponding set integral is:

(24) $$\int f(X)\delta X=\sum_{n\geq 0}\frac{1}{n!}\sum_{(\ell_{1},\ldots ,\ell )\in L_{\infty }^{n}}f(\{(u_{1},\ell_{1}),\ldots ,(u_{n},\ell_{n})\})du_{1}\cdots du_{n}.$$

Let *X =* {(**u**_1_,ℓ_1_), …, (**u***_n_*,ℓ*_n_*)} ⊆ ℑ × *L*_0_. Then the set of labels of the targets in *X* is denoted as *X_L_* = {ℓ_1_, …, ℓ*_n_*}. Given this, *X* is a *labeled multitarget state-set* if |*X_L_*| = |*X*|—i.e., if its elements have distinct labels, in which case targets are uniquely identified. An RFS Ξ ⊆ ℑ × *L*_0_ is a *labeled RFS* (LRFS) if |Ξ*_L_*| = |Ξ| for all realizations Ξ = *X* of Ξ. Consequently, the distribution of an LRFS Ξ has the following property: $f_{\Xi }$(*X*) = 0 if |*X_L_*| ≠ |*X*|. 

In LRFS theory, labels ℓ are *unknown random state variables, which must be Bayes-optimally estimated* along with the unknown random kinematic states **u**_1_, …, **u***_n_*. By way of contrast, in conventional track-management approaches labels are *deterministic, heuristic bookkeeping devices*. 

The LRFS approach requires appropriate definitions of $p_{S}^{k|k-1}(u^{\prime },\ell^{\prime })$, $p_{D}^{k}(u,\ell )$, *f_k_*(**z**|**u**,ℓ) and *f_k|k_*_−1_(**u**,ℓ|**u**′,ℓ′) when (**u**,ℓ), (**u**′,ℓ′) ∈ ℑ × *L*_0_. The primary distinction is that *f_k|k_*_−1_(**u**,ℓ|**u**′,ℓ′) = *δ*_ℓ,ℓ′_·*f_k|k_*_−1_(**u**|**u**′,ℓ′)—i.e., targets do not change labels. For purposes of multitarget tracking and classification (see Remark 4), these quantities will usually depend on the labels. However, for general tracking it can usually be assumed that $p_{S}^{k|k-1}(u^{\prime },\ell^{\prime })=p_{S}^{k|k-1}(u^{\prime }),$$p_{D}^{k}(u,\ell )=p_{D}^{k}(u)$, *f_k_*(**z**|**u**,ℓ) = *f_k_*(**z**|**u**), and *f_k|k_*_−1_(**u**|**u**′,ℓ′) = *f_k|k_*_−1_(**u**|**u**′).

### 3.2. Important Labeled RFSs

These are most simply defined in terms of their PGFLs, where 0 ≤ *h*(**u**,ℓ) ≤ 1 are labeled test functions:

1. *Labeled Multi-Bernoulli (LMB) LRFS*: $G_{\Xi }[h]=\prod_{\ell \in J}^{}\left(1-q_{\ell }+q_{\ell }\cdot s_{\ell }[h]\right)$ where *J* ⊆ *L*_0_ is finite, 0 ≤ *q*_ℓ_ ≤ 1 and *s*_ℓ_[*h*] = ∫*h*(**u**,ℓ)⋅*s*(**x**,ℓ)*d***u** and *s*_ℓ_[1] = 1 for all ℓ ∈ *J*.
2. *Labeled Multi-Bernoulli Mixture* (LMBM) *LRFS*: $G_{\Xi }[h]=\sum_{i=1}^{N}w_{i}\prod_{\ell \in J_{i}}\left(1-q_{\ell }+q_{\ell }\cdot s_{\ell }[h]\right)$.
3. *Generalized Labeled Multi-Bernoulli* (GLMB) *LRFS*: $G_{\Xi }[h]=\sum_{o\in O}\sum_{L\subseteq L_{\infty }}w_{o}(L)\prod_{\ell \in L}s_{o,\ell }[h]$ where: (a) *O* is a finite set of indices *o*; (b) *s_o_*_,ℓ_(**u**) = s*_o_*(**u**,ℓ) with ∫*s_o_*_,ℓ_(**u**)*d***u** = 1 for each *o*,ℓ is the spatial distribution corresponding to the target label ℓ and the index *o*; (c) *ω_o_*(*L*) ≥ 0 for all finite *L* ⊆ *L*_0_; (d) Σ*_o_*_∈_ *_O_* Σ*_L_ω_o_*(*L*) = 1; and (e) *s_o_*_,ℓ_[*h*] = ∫*h*(**u**,ℓ)⋅*s_o_*(**u**,ℓ)*d***u**.

Any labeled multitarget distribution can be approximated by a GLMB distribution that has the same PHD and cardinality distribution [26].

**Remark** **3.** *A Poisson RFS*Ξ ⊆ ℑ × *L*_0_ *is not an LRFS. For, let D*(**u**,ℓ) ≥ 0 *be a PHD on* ℑ *× L*_0_*—i.e., ∫ D*(**u**,ℓ)*d***u** *= 0 for all but a finite number of* ℓ ∈ *L*_0_ *with μ =* Σ_ℓ_ ∫ *D*(**u**,ℓ)*d***u***. Let* $f_{\Xi }$(*X) be Poisson with PHD D*(**u**,ℓ): $f_{\Xi }$(X) = *e*^−^*^μ^*Π_(**u**,ℓ) ∈_ *_X_D*(**u**,ℓ) *and let X*₀ = {(**u**_1_,ℓ₀), …, (**u***_n_*,ℓ₀)} *with |X*₀*| = n. Then, $f_{\Xi }$(X₀) ≠ 0 even though the target with label ℓ₀ has n different kinematic states.*

### 3.3. The GLMB Filter

This filter was introduced in 2011 in [8] and elaborated in [6,7]. Suppose that *L*_0_ = {0,1, …,} × {1,…} and if ℓ = (*k*,*i*) then *t_k_* is the time that trackℓ was initiated and *i* ≥ 1 distinguishes it from any other track created at time *t_k_*. At time *t_k_*, a finite number of labels in *L_k_*= {*k*} × {1, …} are assigned to hypothesized newly-appearing tracks. Thus, at time *t_k_*, the set *L*_[0:*k*]_ of all currently assigned track labels is a finite subset of *L*_0:*k*_ = {0,1, …, *k*} × {1,…}; and each such label is an unknown discrete random variable ℓ ∈ *L*_[0:*k*]_ which must be estimated.

Given this, the family of GLMB distributions solves the labeled multitarget Bayes filter in exact closed form. In particular, at time *t_k_*:

(25) $$f_{k|k}(X|Z_{1:k})=\delta_{\left|X|,|X_{L}\right|}\sum_{\left(\alpha_{1},\ldots ,\alpha_{k}\right)}\begin{array}{c} \omega_{\alpha_{1},\ldots ,\alpha_{k}}^{k|k}(X_{L})\cdot {\left(s_{\alpha_{1},\ldots ,\alpha_{k}}^{k|k}\right)}^{X} \end{array}$$

where the summation is taken over all (*α*_1_,…,*α_k_*) ∈ *A*_1_ × … × *A_k_*; where each *α_i_*:*L*_[0:*i*]_ → {0,1, …, |*Z_i_*|} is a label-to-measurement association—i.e., if *α_i_*(ℓ) = *α_i_*(ℓ′) > 0 then ℓ = ℓ′; where $s_{\alpha_{1},\ldots ,\alpha_{k}}^{k|k}(u,\ell )$ is a target spatial distribution; and where *A_i_* denotes the set of all such associations *α_i_* at time *t_k_*.

The GLMB filter is a *true Bayesian multitarget tracker* because it is guaranteed to propagate target tracks with unique track labels (a “true” tracker), which in turn are realizations of unknown random identity-variables (a “Bayesian” tracker). 

Moreover, because it is an exact closed-form solution of the labeled multitarget Bayes filter, the GLMB filter has *provably Bayes-optimal track-management*. At time *t_k−_*_1_, an (approximate) Bayes-optimal multitarget state estimate *X_k−_*_1*|k−*1_ is extracted from *f_k−_*_1*|k−*1_(*X*|*Z*_1:*k−*1_). At time *t_k_*, a similar estimate *X_k|k_* is extracted from *f_k|k_*(*X*|*Z*_1:*k*_). If (**u**,ℓ) ∈ *X_k−_*_1*|k−*1_ and (**u**′,ℓ) ∈ *X_k|k_* then (**u**,ℓ) and (**u**′,ℓ) both belong to the track with label ℓ. If (**u**′,ℓ) ∉ *X_k|k_* for any **u**′ then track ℓ has been dropped. If (**u**,ℓ) ∈ *X_k|k_* but (**u**′,ℓ) ∉ *X_k−_*_1*|k−*1_ for any **u**′ then a track with label ℓ has been initiated or reacquired.

Due to the number of association-vectors (*α*_1_, …, *α_k_*) increases without bound, the summation in Equation (25) must be pruned at every time-step. The information loss due to pruning can be characterized exactly—i.e., the *L*_1_ norm between the pruned and unpruned distributions is the sum of the weights of the pruned terms [6] (Proposition 5).

Using Gibbs stochastic sampling techniques, the GLMB filter can be implemented with computational order *O*(*n*^2^*m*) where *m* is the current number of measurements and *n* the current number of tracks [5]. This is particularly advantageous when clutter is dense. The most recent such implementations can simultaneously track over a million 2D targets in significant clutter using off-the-shelf computing equipment [27].

**Remark** **4.** *Every target has a unique identity state variable [25] (pp. 135, 196–197). A track label is a provisional identity assigned to a target in lieu of its actual identity. The GLMB filter can be generalized from joint multitarget detection and tracking to joint multitarget detection, tracking, and identification. This is accomplished by incorporating identity information into target labels [9]*.

### 3.4. The LMBM Filter

In [10] it was shown that the family of LMBM distributions solves the labeled multitarget Bayes filter in exact closed form. The corresponding LMBM filter is, therefore, a true Bayesian multitarget tracker with provably Bayes-optimal track management. It is somewhat less computationally expensive than the GLMB filter, but also less accurate since LMBM distributions are less accurate approximations of labeled multitarget distributions than GLMB distributions.

## 4. The PMBM Filter

There are at least three successive versions of the PMBM filter. The section is organized as follows: the “unlabeled” PMBM (U-PMBM) filter (Section 4.1); the “undetected targets” interpretation of this filter (Section 4.2); the “label-augmented” PMBM (LA-PMBM) filter (Section 4.3); “hybrid labeled-unlabeled” RFSs (Section 4.4); the “hybrid labeled-unlabeled” PMBM (H-PMBM) filter (Section 4.5); and theoretical issues with the H-PMBM filter (Section 4.6).

### 4.1. Unlabeled PMBM (U-PBMB) Filter

In this original 2012 version [11], all RFSs are unlabeled—LRFSs are never mentioned. All target-birth RFSs are assumed to be Poisson—in our notation, $G_{B}^{k|k-1}[h]=e^{D_{B}^{k|k-1}[h-1]}$ for all *k* ≥ 1—as is the initial RFS: $G_{0|0}[h]=e^{D_{0|0}[h-1]}$. Given this, the PMBM filter propagates PMBM distributions in exact closed form. Specifically, $G_{k|k-1}[h]=F_{k|k-1}^{u}[h]\cdot F_{k|k-1}^{d}[h]$ and $G_{k|k}[h]=F_{k|k}^{u}[h]\cdot F_{k|k}^{d}[h]$ where $F_{k|k-1}^{u}[h]=e^{D_{k|k-1}[h-1]}$ and $F_{k|k}^{u}[h]=e^{D_{k|k}[h-1]}$ are Poisson and where $F_{k|k-1}^{d}[h]$ and $F_{k|k}^{d}[h]$ are MBM. The demonstration of this fact in [11,12] is somewhat sketchy. The following PGFL-based verification of it will be useful in the sequel.

**U-PMBM Filter Time-Update**. According to Equation (20) and substituting $G_{B}^{k|k-1}[h]=e^{D_{B}[h-1]}$, the PGFL prediction formula is:

(26) $$G_{k|k-1}[h]=e^{D_{B}[h-1]}\cdot G_{k-1|k-1}[1+p_{S}M_{h-1}]$$

where, by assumption, *G_k_*_−1|*k*−1_[*h*] is PMBM:

(27) $$G_{k-1|k-1}[h]=e^{D[h-1]}\sum_{l=1}^{\nu }w_{l}\prod_{i=1}^{N_{l}}(1+q_{l,i}\cdot s_{l,i}[h-1]).$$

Thus, predicted PGFL is easily seen to be PMBM:

(28) $$G_{k|k-1}[h]=e^{D_{k|k-1}[h-1]}\sum_{l=1}^{\nu }w_{l}\prod_{i=1}^{N_{l}}\left(1+{q^{\prime }}_{l,i}\cdot {s^{\prime }}_{l,i}[p_{S}M_{h-1}]\right)$$

where:

(29) $$D_{k|k-1}(x)=D_{B}(x)+D[p_{S}M_{x}]=D_{B}(x)+\int p_{S}(x^{\prime })\cdot f_{k|k-1}(x|x^{\prime })\cdot D(x^{\prime })dx^{\prime }$$

(30) $${q^{\prime }}_{l,i}=q_{l,i}\cdot s_{l,i}[p_{S}],\begin{array}{ccc} & & {s^{\prime }}_{l,i}(x)=\frac{s_{i,i}[p_{S}M_{x}]}{s_{l,i}[p_{S}]}=\frac{\int p_{S}(x^{\prime })\cdot f_{k|k-1}(x|x^{\prime })\cdot s_{l,i}(x^{\prime })dx^{\prime }}{\int p_{S}(x^{\prime })\cdot s_{l,i}(x^{\prime })dx^{\prime }}. \end{array}$$

**U-PMBM Filter Measurement-Update**. Let *Z =* {**z**_1_, …, z*_m_*} with |Z| = *m* be collected at time ***t****_k_*. According to Equation (21) the measurement-updated PGFL is:

(31) $$G_{k|k}[h|Z_{1:k}]=\frac{\frac{\delta F}{\delta Z}[0,h]}{\frac{\delta F}{\delta Z}[0,1]}$$

where, by Equation (22), *F*[*g*,*h*] = *e^κ^*^[*g*−1]^⋅*G_k_*_|*k*−1_[*h*(1 + *p_D_L_g_*_−1_)] and, by assumption, *G_k_*_|*k*−1_[*h*] is PMBM:

(32) $$G_{k|k-1}[h]=e^{D[h-1]}\sum_{l=1}^{\nu }w_{l}\prod_{i=1}^{N_{l}}(1+q_{l,i}s_{l,i}[h-1]).$$

Thus, $F[g,h]=\sum_{l=1}^{\nu }w_{l}\cdot F_{l}[g,h]$, where:

(33) $$F_{l}[g,h]=e^{\kappa [g-1]+D[h(1+p_{D}L_{g-1})-1]}\prod_{i=1}^{N_{l}}\left(1+q_{l,i}s_{l,i}[h(1+p_{D}L_{g-1})-1]\right)$$

and the measurement-updated PGFL is:

(34) $$G_{k|k}[h]=\frac{\sum_{l=1}^{\nu }w_{l}\cdot \frac{\delta F_{l}}{\delta Z}[0,h]}{\sum_{l=1}^{\nu }w_{l}\cdot \frac{\delta F_{l}}{\delta Z}[0,1]}=\sum_{l=1}^{\nu }{\tilde{w}}_{l}\cdot G_{l}[h]$$

where:

(35) $${\tilde{w}}_{l}=\frac{w_{l}\cdot \frac{\delta F_{l}}{\delta Z}[0,1]}{\sum_{l=1}^{\nu }w_{l}\cdot \frac{\delta F_{l}}{\delta Z}[0,1]},G_{l}[h]=\frac{\frac{\delta F_{l}}{\delta Z}[0,h]}{\frac{\delta F_{l}}{\delta Z}[0,1]}.$$

Since each *F_l_*[*g*,*h*] has the same Poisson factor $e^{\kappa [g-1]+D[h(1+p_{D}L_{g-1})-1]}$, it is sufficient to show that the measurement-update of a PMB PGFL is a PMBM PGFL Accordingly, in what follows we neglect the index *l* in *F_l_*[*g*,*h*].

Two cases must be considered: *N* = 0 and *N* > 0. If the former, then $F[g,h]=e^{\kappa [g-1]+D[h(1+p_{D}L_{g-1})-1]}$ and so from the chain rule for functional derivatives [14] (Equation (11.280)), we find that *G_k_*_|*k*_[*h*] is PMB:

(36) $$G_{k|k}[h]=e^{D[(h-1)(1-p_{D})]}\prod_{z\in Z}\frac{\kappa (z)+D[hp_{D}L_{z}]}{\kappa (z)+D[p_{D}L_{z}]}.$$

Now assume *N* ≥ 1. Applying the general product rule for functional derivatives [14] (Equation (11.274)) to Equation (33):

(37) $$\frac{\delta F}{\delta Z}[g,h]=\sum_{W_{0}\cup W_{1}\cup \ldots \cup W_{N}=Z}\begin{array}{c} \left(\frac{\delta }{\delta W_{0}}e^{\kappa [g-1]+D[h(1+p_{D}L_{g-1})-1]}\right)\prod_{i=1}^{N}\frac{\delta }{\delta W_{i}}\left(1+q_{i}s_{i}[h(1+p_{D}L_{g-1})-1]\right) \end{array}$$

(38) $$=e^{\kappa [g-1]+D[h(1+p_{D}L_{g-1})-1]}\left(\prod_{i=1}^{N}\left(1+q_{i}s_{i}[h(1+p_{D}L_{g-1})-1]\right)\right)\sum_{W_{0}\cup W_{1}\cup \ldots \cup W_{N}=Z}\theta^{W_{0}}\begin{array}{cc} \prod_{i=1}^{N}\frac{\frac{\delta }{\delta W_{i}}\left(1+q_{i}s_{i}[h(1+p_{D}L_{g-1})-1]\right)}{1+q_{i}s_{i}[h(1+p_{D}L_{g-1})-1]} & \end{array}$$

where *θ*(**z**) = *k*(**z**) + *D*[*hp_D_L***_z_**]. The *i*-th fraction in the rightmost product is nonzero only if *W_i_* is empty or a singleton; and if *W_i_* = ∅ then it is 1. Thus, each list *W*_0_, *W*_1_, …, *W_N_* is mathematically equivalent to an association *α*:{1, …, *N*}→{0,1, …, *m*}—*i* (i.e., *α*(*i*) = *α*(*i*′) > 0 implies *I* = *i*′. Setting *g* = 0, Equation (38) becomes, after some algebraic manipulations:

(39) $$\frac{\delta G_{k|k}}{\delta Z}[0,h]=e^{-\kappa [1]+D[h(1-p_{D})-1]}\theta^{Z}\sum_{\alpha }\left(\prod_{i:\alpha (i)=0}\left(1+q_{i}s_{i}[h(1-p_{D})-1]\right)\right)\left(\prod_{i:\alpha (i)>0}\frac{q_{i}s_{i}[hp_{D}L_{z_{\alpha (i)}}]}{\theta (z_{\alpha (i)})}\right)$$

where the summation is taken over all associations. Therefore, the measurement-updated PGFL for a PMB predicted PGFL is:

(40) $$G_{k|k}[h]=e^{D[(h-1)(1-p_{D})]}\cdot \frac{\sum_{\alpha }\left(\prod_{i:\alpha (i)=0}\left(1+q_{i}s_{i}[h(1-p_{D})-1]\right)\right)\left(\prod_{i:\alpha (i)>0}\frac{q_{i}s_{i}[hp_{D}L_{z_{\alpha (i)}}]}{\theta (z_{\alpha (i)})}\right)}{\sum_{\alpha }\left(\prod_{i:\alpha (i)=0}\left(1-q_{i}s_{i}[p_{D}]\right)\right)\left(\prod_{i:\alpha (i)>0}\frac{q_{i}s_{i}[p_{D}L_{z_{\alpha (i)}}]}{\theta (z_{\alpha (i)})}\right)}.$$

This can be rewritten as the PMBM PGFL:

(41) $$G_{k|k}[h]=e^{D_{k|k}[h-1]}\sum_{\alpha }w_{\alpha }\left(\prod_{i:\alpha (i)=0}\left(1+{q^{\prime }}_{i}{s^{\prime }}_{i}[h-1]\right)\right)\left(\prod_{i:\alpha (i)>0}{\tilde{s}}_{\alpha ,i}[h]\right)$$

where:

(42) $$D_{k|k}(x)=(1-p_{D}(x))\cdot D(x),\begin{array}{c} {q^{\prime }}_{i}=\frac{q_{i}s_{i}[1-p_{D}]}{1-q_{i}s_{i}[p_{D}]} \end{array}$$

(43) $${s^{\prime }}_{i}(x)=\frac{\left(1-p_{D}(x))\cdot s_{i}(x\right)}{s_{i}[1-p_{D}]},\begin{array}{c} {\tilde{s}}_{\alpha ,i}(x)=\frac{p_{D}(x)\cdot L_{z_{\alpha (i)}}(x)\cdot s_{i}(x)}{s_{i}[p_{D}L_{z_{\alpha (i)}}]} \end{array}$$

(44) $$w_{\alpha }=\frac{\left(\prod_{i:\alpha (i)=0}\left(1-q_{i}s_{i}[p_{D}]\right)\right)\left(\prod_{i:\alpha (i)>0}\frac{q_{i}s_{i}[p_{D}L_{z_{\alpha (i)}}]}{\theta (z_{\alpha (i)})}\right)}{\sum_{\alpha }\left(\prod_{i:\alpha (i)=0}\left(1-q_{i}s_{i}[p_{D}]\right)\right)\left(\prod_{i:\alpha (i)>0}\frac{q_{i}s_{i}[p_{D}L_{z_{\alpha (i)}}]}{\theta (z_{\alpha (i)})}\right)}.$$

### 4.2. Undetected-Target Interpretation of the U-PMBM Filter

The PMBM filter therefore, solves the unlabeled multitarget Bayes filter in exact closed form. However, the PMBM approach goes beyond this to adopt a specific physical interpretation of PMBM RFSs. Let:

(45) $$G_{k|k}^{UP}[h]=e^{D_{k|k}[h-1]}\sum_{l=1}^{N_{k|k}}w_{l}^{k|k}\prod_{i=1}^{N_{l}^{k|k}}\left(1+q_{l,i}^{k|k}s_{l,i}^{k|k}[h-1])=F_{k|k}^{u}[h]\cdot F_{k|k}^{d}[h\right]$$

be the PMBM PGFL at time *t_k_*. It is clear from Equations (29) and (41) that the time- and measurement-updates for the Poisson factors are, respectively, $D_{k|k-1}(x)=D_{B}^{k|k-1}(x)+D_{k-1|k-1}[p_{S}^{k|k-1}M_{x}^{k|k-1}]$ and $D_{k|k}(x)=(1-p_{D}^{k}(x))\cdot D_{k-1|k}(x)$. The formulas for *D_k_*_|*k*−1_(**x**) and *D_k_*_|*k*_(**x**) thus involve $p_{S}^{i|i-1}$, $1-p_{D}^{i}$, and $M_{x}^{i|i-1}$, but not $p_{D}^{i}$, $L_{z}^{i}$, *κ_i_*, *Z*_1:*i−*1_,*Z*_1:*i*_. 

This fact has led to the interpretation of $F_{k|k}^{u}[h]=e^{D_{k|k}[h-1]}$ as a model of the “undetected targets” at time *t_k_* [4,11,12]. According to [11] (p. 1103), these are “…targets that have never been detected”—i.e., not detected at times *t*_1_, …, *t_k_*. It was subsequently stipulated that “…detected targets cannot become undetected targets” [4] (p. 246). 

The primary justification for the PMBM approach is the following: “One significant benefit of the inclusion of a Poisson component is in initialization of the tracker…The Poisson distribution provides a convenient mechanism for specifying a prior distribution on the number and position of targets when little information is available” [12] (p. 1670). 

However, this potential advantage is negated by a major theoretical obstacle: Poisson RFSs require non-unique labels and so are not LRFSs (see Remark 3). Due to this, they cannot be used in any theoretically rigorous, true multitarget tracker.

A more subtle obstacle is this: *it is theoretically impossible to prune PMBM distributions in a practically useful manner*. When a GLMB distribution (Equation (25)) is pruned, the pruned distribution is a GLMB distribution. When a PMBM distribution is pruned, however, *it is usually not even a multitarget density function*. First consider an LMB distribution $f_{\Xi }$({**x**_1_, …, **x***_n_*}) [14] (Equation (11.133)). Any term in it has the form $f_{i_{1},\ldots ,i_{n}}(x_{1},\ldots ,x_{n})\propto f_{i_{1}}(x_{1})\cdots f_{i_{n}}(x_{n})$ where **x**_1_, …, **x***_n_* are distinct, 1 ≤ *i*_1_ ≠ … ≠ *i_n_* ≤ *ν*, and *f*_1_(**x**), …, *f_ν_*(**x**) are distinct density functions. Since $f_{i_{1},\ldots ,i_{n}}(x_{1},\ldots ,x_{n})$ is not symmetric in **x**_1_, …, **x***_n_*—and therefore, not a multitarget density—neither is any other pruning of $f_{\Xi }$. Now, let $f_{\Xi }$ be a PMB distribution:

(46) $$f_{\Xi }(X)=e^{-D[1]}D^{X}\left(\prod_{i=1}^{\nu }\left(1-q_{i}\right)\right)\sum_{\alpha }\prod_{i:\alpha (i)>0}\frac{q_{i}\cdot s_{i}(x_{\alpha (i)})}{\left(1-q_{i})\cdot D(x_{\alpha (i)}\right)}$$

where *α*: {1, …, *ν*} → {0, 1, …, *n*} is an association. Its terms have the same form as before, except that the *f_i_*can be equal to *D* but those *f_i_*that are not *D* are distinct. Since $f_{i_{1},\ldots ,i_{n}}$ is symmetric only when *f_j_*= *D* for all *j* = 1, …, *n*, no pruning of $f_{\Xi }$ other than this case is a multitarget density. What *is* theoretically permissible is to prune an MBM (resp. PMBM) PGFL by eliminating one or more of its MB (resp. PMB) PGFL terms. However, pruning the individual terms of the corresponding MB (resp. PMB) distributions is not permissible—which is exactly what is required to eliminate specific small-weight hypotheses.

### 4.3. “Label-Augmented” PMBM (LA-PMBM) Filter

As was noted at the beginning of Section 3.1, unlabeled RFS-based filters, such as the GM-CPHD filter, can heuristically propagate tracks even though they are not true multitarget trackers. The U-PMBM filter can propagate tracks using similar heuristics, but it—like the GM-CPHD filter—is not a true multitarget tracker since it is unlabeled. Accordingly, in 2015 it was modified as follows: “… [the Vo-Vo paper [7]] shows that the labelled case can be handled within the unlabeled framework by incorporating a label element in to the underlying state space” [12] (p. 1675). That is, it was claimed that the PMBM filter can be extended to the labeled case by replacing the unlabeled single-target state space ℑ with the labeled state space ℑ × *L*_0_.

This modified PMBM filter will be referred to as the “label-augmented” PMBM (LA-PMBM) filter. It must propagate PMBM RFSs of ℑ × *L*_0_ with PGFLs:

(47) $$G_{k|k}^{LAP}[h]=e^{D_{k|k}[h-1]}\sum_{l=1}^{N_{k|k}}w_{l}^{k|k}\prod_{i=1}^{N_{l}^{k|k}}(1+q_{l,i}^{k|k}s_{l,i}^{k|k}[h-1])=F_{k|k}^{u}[h]\cdot F_{k|k}^{d}[h].$$

Now, however, the PHD *D_k_*_|*k*_ and spatial distributions $s_{l,i}^{k|k}$ must have the respective forms *D_k_*_|*k*_(**u**,ℓ) and $s_{l,i}^{k|k}(u,\ell )$, where ∫*D_k_*_|*k*_ (**u**,ℓ)*d***u** = 0 and ∫$s_{l,i}^{k|k}(u,\ell )du=0$ for all but a finite number of ℓ and where $s_{l,i}^{k|k}[1]=\sum_{\ell }\int s_{l,i}^{k|k}(u,\ell )du=1$ for all *l,i*.

There is a serious theoretical difficulty, however: the $s_{l,i}^{k|k}$ *are not track distributions*. For if otherwise, $\int s_{l,i}^{k|k}(u,\ell )du=1$ would imply that $s_{l,i}^{k|k}[1]>1$, a contradiction. Therefore, $s_{l,i}^{k|k}$ appears to be physically meaningless. 

Beyond this, the above claim—that “the labelled case can be handled within the unlabeled framework”—is untrue. As was noted in Remark 3, a Poisson RFS on ℑ × *L*_0_ is not an LRFS since it *requires* nondistinct target labels. Consequently, it is not possible for the LA-PMBM filter to be a true multitarget tracker. Instead, it “…is able to maintain track continuity implicitly based on the information provided by metadata” [4] (p. 245)—that is, only heuristically.

### 4.4. “Hybrid Labeled-Unlabeled” RFSs

Like the U-PMBM filter, the LA-PMBM filter is not a true multitarget tracker—a fact that was pointed out in 2017 in [23] (Section XI-E). Apparently to address this issue, it was modified in 2018 as follows [4] (p. 246): A single common label—ℓ*, say—is assigned to all “undetected targets” at all times, whereas “detected targets” are uniquely labeled as in LRFS theory. Additionally, the “undetected-target” RFS at any time is assumed to be a Poisson RFS on ℑ × {ℓ*} (a slightly later paper, [3], also appears to employ the H-PMBM approach, except that ℓ* is implicit rather than explicit.) 

No careful theoretical foundation for the hybrid approach was provided in [4]. It is, therefore, necessary to construct one here. The label space is *L*_0_ = *L*^\^*∪{ℓ*} where *L*^\^* = *L*_0_ − {ℓ*}. Given a finite subset *X* = {(**u**_1_,ℓ_1_), …, (**u***_n_*,ℓ*_n_*)} ⊆ ℑ × *L*_0_, as usual let *X_L_*= {ℓ_1_, …, ℓ*_n_*} denote the set of labels in *X*. Additionally, let X* = {(**u**,ℓ) ∈ *X*|ℓ = ℓ*} be the subset of *X* of targets that are “undetected”; and let *X*^\^* = *X* − X* be the targets in *X* that are “detected.” Then it is assumed that the only legitimate state-sets *X* are those such that |*X*^\^*| = |*X_L_* − {ℓ*}|—i.e., those for which the detected targets have distinct labels other than ℓ*. Let us refer to these as “hybrid state-sets.” Let Ξ be a “hybrid RFS”—i.e., an RFS of ℑ × *L*_0_ whose instantiations are hybrid. Then it must be the case that $f_{\Xi }$ (*X*) = 0 if *X* is not hybrid. Thus, every distribution defined for hybrid *X* must include the factor $\delta_{\left|X^{\backslash *}|,|X_{L}-\{\ell^{*}\}\right|}$.

The goal of Sections XI-XIII of [4] is to apply the PMBM filter, Equations (28) and (41), to the hybrid state space ℑ × *L*_0_. This will be addressed in the next section. First, however, we must reformulate $p_{S}^{k|k-1}(u^{\prime },\ell^{\prime })$, $p_{D}^{k}(u,\ell )$, *f_k_*(**z**|**u**,ℓ), $D_{B}^{k|k-1}(u,\ell )$, and *f_k|k-_*_1_(**u,**ℓ|**u**′,ℓ′) when (**u**,ℓ), (**u**′,ℓ′) ∈ ℑ × *L*_0_ are hybrid. For “detected targets” (i.e., ℓ ≠ ℓ* and ℓ′ ≠ ℓ*), the usual LRFS formulation applies. For “undetected targets,” it is reasonable to define $p_{S}^{k|k-1}(u^{\prime },\ell^{*})=p_{S}^{k|k-1}(u^{\prime })$, $p_{D}^{k}(u,\ell^{*})=p_{D}^{k}(u),$ and *f_k_*(**z**|**u**,ℓ*) = *f_k_*(**z**|**u**). It also makes sense to define $D_{B}^{k|k-1}(u,\ell )=\delta_{\ell ,\ell^{*}}\cdot D_{B*}^{k|k-1}(u)$ for some $D_{B*}^{k|k-1}(u)$ since, by definition, targets that have just appeared cannot have been detected yet (to wit: “…these states of…newborn targets will be described by…a Poisson RFS…[whose] elements have label [ℓ*]”[4] (p. 247).

Finally, consider *f_k|k−_*_1_(**u,**ℓ|**u**′,ℓ′) = *p_k|k−_*_1_(ℓ|**u**′,ℓ′)⋅*f_k|k−_*_1_(**u**|**u**′,ℓ,ℓ′) when ℓ = ℓ* or ℓ′ = ℓ*. “Undetected targets” must retain the label ℓ*, since they cannot become “detected targets” in the absence of a detection process that has not yet occurred, and if ℓ′ ≠ ℓ* then *p_k|k−_*_1_(ℓ*|**u**′,ℓ′) = 0 since “…detected targets cannot become undetected targets” [4] (p. 246). Thus, *p_k|k−_*_1_(ℓ|**u**′,ℓ*) = *δ*_ℓ,ℓ*_⋅*p_k|k−_*_1_(ℓ*|**u**′,ℓ*), in which case it is reasonable to assume that *p_k|k−_*_1_(ℓ*|**u**′,ℓ*) = *p_k|k−_*_1_(ℓ*|ℓ*) = 1 and *f_k|k−_*_1_(**u**|**u**′,ℓ*,ℓ*) = *f_k|k−_*_1_(**u**|**u**′). (Note that if target identity is to be taken into account as per Remark 4, these simplifications are no longer appropriate.)

### 4.5. “Hybrid Labeled-Unlabeled” PMBM (H-PMBM) Filter

Now let:

(48) $$G_{k|k}^{HP}[h]=e^{D_{k|k}[h-1]}\sum_{l=1}^{N_{k|k}}w_{l}^{k|k}\prod_{\ell \in L_{l}^{k|k}}^{}\left(1+q_{l,\ell }^{k|k}s_{l,\ell }^{k|k}[h-1])=F_{k|k}^{u}[h]\cdot F_{k|k}^{d}[h\right]$$

be the PGFL of a PMBM RFS Ξ on ℑ × *L*_0_ with ℓ* ∈ *L*_0_, where the MBM factor in Equation (45) has been replaced by an LMBM factor since “detected targets” are now assumed to be uniquely labeled; and where $L_{l}^{k|k}$ are finite subsets of *L*_0_ and $\int s_{l,\ell }^{k|k}(u)du=\int s_{l}^{k|k}(u,\ell )du=1$ for all $\ell \in L_{l}^{k|k}$. Since the Poisson factor $F_{k|k}^{u}[h]=e^{D_{k|k}[h-1]}$ applies only to “undetected targets” with common label ℓ*, it must be the case that $\ell^{*}\notin L_{l}^{k|k}$ for every *l* = 1,..,*N_k_*_|*k*_ and that $D_{k|k}(u,\ell )=\delta_{\ell ,\ell^{*}}\cdot D_{k|k}^{*}(u)$ for some $D_{k|k}^{*}(u)$. We will refer to Equation (48) as an “H-PMBM PGFL”.

Given this, $f_{k|k}^{d}(X^{d}|Z_{1:k})$ and $f_{k|k}^{u}(X^{u}|Z_{1:k})$ “….can be propagated in parallel, in both cases by carrying out a prediction step and an update step…” [4] (p. 248); where the undetected-target distribution $f_{k|k}^{u}(X^{u}|Z_{1:k})$ with *X^u^*⊆ ℑ × {ℓ*} is Poisson and the detected-target distribution $f_{k|k}^{d}(X^{d}|Z_{1:k})$ with *X^d^* ⊆ ℑ × (*L*_0_ − {ℓ*}) is LMBM. 

Moreover, the following claim is made about these two filters: “In the following development of the prediction and update steps, we use the fact that the posterior pdf of the overall multitarget state RFS…factorizes as…” (in current notation):

(49) $$f_{k|k}(X|Z_{1:k})=f_{k|k}^{d}(X^{d}|Z_{1:k})\cdot f_{k|k}^{u}(X^{u}|Z_{1:k}).$$

However, Equation (49) is untrue. For by Bayes’ rule:

(50) $$f_{k|k}(X|Z_{1:k})=f_{k|k}(X^{d},X^{u}|Z_{1:k})=f_{k|k}^{d}(X^{d}|X^{u},Z_{1:k})\cdot f_{k|k}^{u}(X^{u}|Z_{1:k})$$

It then follows from Equation (49) that $f_{k|k}^{d}(X^{d}|X^{u},Z_{1:k})=f_{k|k}^{d}(X^{d}|Z_{1:k})$. Likewise, $f_{k|k}^{u}(X^{u}|X^{d},Z_{1:k})=f_{k|k}^{u}(X^{u}|Z_{1:k}).$ Thus, RFSs *X^u^*and *X^d^* are statistically independent of each other. This means that the filter for $f_{k|k}^{d}(X^{d}|Z_{1:k})$ and the filter for $f_{k|k}^{u}(X^{u}|Z_{1:k})$ are *statistically decoupled*. However, this is not the case, since “…the update step for *X^d^* involves the prediction results for both *X^d^* and *X^u^*…” [4] (p. 248). (This is because the LMBM component of *G_k_*_|*k*_[*h*|*Z*_1:*k*_] in Equation (41) depends on the Poisson component $e^{D_{k|k-1}[h-1]}$ of *G_k_*_|*k*−1_[*h*|*Z*_1:*k*−1_] via *θ_k_*(**z**) = *κ_k_*(**z**)+*D_k_*_|*k*−1_[*hp_D_L***_z_**]. That is, $f_{k|k}^{d}(X^{d}|X^{u},Z_{1:k})\neq f_{k|k}^{d}(X^{d}|Z_{1:k})$.)

### 4.6. Theoretical Issues With the H-PMBM Filter

From Section 4.1 we know that the PMBM filter on ℑ × *L*_0_ is guaranteed to propagate PMBM distributions on ℑ × *L*_0_ in exact closed form. However, does it propagate *hybrid* PMBM distributions in exact closed form? This does not appear to be the case. 

Consider, for example, Equation (36) with *k* = 1 and with the single-target state space being ℑ × *L*_0_ (with ℓ*∈ *L*_0_) rather than ℑ:

(51) $$G_{1|1}^{HP}[h]=e^{D_{1|1}[h-1]}\prod_{z\in Z_{1}}\left(1-q_{z}^{1|1}+q_{z}^{1|1}\cdot \frac{D_{B}^{1|0}[hp_{D}^{1}L_{{}_{z}}^{1}]}{D_{B}^{1|0}[p_{D}^{1}L_{z}^{1}]}\right)=e^{D_{1|1}[h-1]}\prod_{z\in Z_{1}}\left(1-q_{z}^{1|1}+q_{z}^{1|1}\cdot s_{z}^{1|1}[h]\right).$$

Here, $D_{B}^{1|0}[p_{D}^{1}L_{z}^{1}]=D_{B*}^{1|0}[p_{D}^{1}L_{z}^{1}]$ since $p_{D}^{1}$ and $L_{z}^{1}$ are independent of labels, and:

(52) $$D_{1|1}(u,\ell )=(1-p_{D}^{1}(u))\cdot D_{B}^{1|0}(u,\ell )$$

(53) $$q_{z}^{1|1}=\frac{D_{B*}^{1|0}[p_{D}^{1}L_{z}^{1}]}{\kappa_{1}(z)+D_{B*}^{1|0}[p_{D}^{1}L_{z}^{1}]},\begin{array}{cccc} & & s_{z}^{1|1}(u,\ell )=\frac{p_{D}^{1}(u)\cdot L_{z}^{1}(u)\cdot D_{B}^{1|0}(u,\ell )}{D_{B*}^{1|0}[p_{D}^{1}L_{z}^{1}]}. & \end{array}$$

Given that $D_{B}^{1|0}(u,\ell )=\delta_{\ell ,\ell^{*}}\cdot D_{B*}^{1|0}(u)$, *D*_1|1_(**u**,ℓ) has the correct form for an H-PMBM PGFL However, *the product is not an LMB PGFL* This is because the individual Bernoulli factors are indexed by the elements of *Z*_1_, not by labels in *L*_0_. 

Thus, as a heuristic workaround, a distinct label ℓ**_z_**≠ ℓ* is assigned to each **z** ∈ *Z*_1_: “For each measurement…a new Bernoulli component is created, to which [a] unique label…is assigned” [4] (following Equation (107)); and: “…[e]ach measurement at each time step gives rise to a new potentially detected target. That is, there is the possibility that a new measurement is the first detection of a target, but it can also correspond to another previously detected target or clutter, in which case there is no new target. As this target may exist or not, its resulting distribution is Bernoulli and we refer to it as [sic] ‘potentially detected target’” [3] (p. 1885). 

It follows that the labeled track distribution of the Bernoulli representation of the “potentially detected target” corresponding to **z**∈ *Z*_1_ must be $s^{1|1}(u,\ell_{z})=s_{z}^{1|1}(u,\ell_{z})$.

This workaround results in at least three theoretical difficulties:

1. There is an inherent theoretical conflict between labeling using ℓ∈ *L*_0_ and labeling using **z**∈ *Z*_1_. Since $s_{z}^{1|1}(u,\ell_{z})\propto D_{B}^{1|0}(u,\ell_{z})$ and since $D_{B}^{1|0}(u,\ell )=0$ if ℓ ≠ ℓ* and since ℓ**_z_** ≠ ℓ*, it follows that *s*^1|1^(**u**,ℓ**_z_**) = 0 identically for all **z** ∈ *Z*_1_—a contradiction. One could sidestep this difficulty by redefining $s_{z}^{1|1}(u,\ell_{z})\propto p_{D}^{1}(u)\cdot L_{z}^{1}(u)\cdot D_{B*}^{1|0}(u,\ell )$, but this would be another heuristic workaround.
2. In [4] (pp. 245–246) the following was stated: “In cases of limited prior birth information, one typically uses a heuristic to generate new Bernoulli components based on measurements from the previous time step (Reuter et al. [28]). Such heuristics can be avoided with the MB-Poisson model…” This is untrue on both counts. First, and as was noted following Equation (17), approaches that dynamically estimate the target-birth process “based on measurements from the previous time step”—i.e., based on *Z_k_*_-1_—are theoretically permissible. Examples include [29] and [30]—and [28]. Second, note that the “MB-Poisson model” employs a “heuristic to generate new Bernoulli components based on measurements from”: the current time-step! Thus, how is it conceptually different from the approach in [28]?
3. More seriously, the dynamical transition of undetected targets to detected ones occurs during the measurement-update, as mediated by *f_k_*(*Z_k_*|*X*), rather than—as theoretically should be the case—during the time-update, as mediated by *f_k|k−_*_1_(*X*|*X*′,*Z*_1:*k−*1_). Thus, *f_k_*(*Z_k_*|*X*) has been implicitly assumed to have the form *f_k_*(*Z_k_*|*X*,*X*′)—which is not the case (see Equation (54)).

The H-PMBM filter therefore, does not appear to have a theoretically rigorous, closed-form mechanism for assigning labels to newly-detected “undetected targets.” And this fact is a direct consequence of the Poisson factor in Equation (48).

However, there is a far more fundamental theoretical and phenomenological difficulty: *the hybrid approach has no basis in physical reality*. Targets are physically real entities regardless of whether or not they are detected. They have distinct (but unknown) real-world identities and therefore, inherently have distinct (unknown) labels. As was noted in Remark 4, target labels in *L*_0_ are provisional identities assigned in lieu of more precise identifying information. LRFS labels are, therefore, not “artificial variables that are added to the target states” [3] (p. 1884). Rather, they are *standbys for the realizations of a physically real random state-variable: target identity*. The H-PMBM approach, by way of contrast, requires targets with label ℓ* to have multiple kinematic states simultaneously—a *physical impossibility*.

## 5. A Statistical Theory of Undetected Targets

The meaning of the “undetected target” concept is extremely unclear. Thus, the purpose of this section is to devise a statistically rigorous—and yet intuitive—theory of undetected targets. As stated in the Introduction, our ultimate goal is to construct a concrete formula for the posterior “undetected targets” PGFL $G_{k|k}^{u}[h|Z_{1:k}]$. The argument presented is as follows.

1. Section 5.1: We surmise that the “undetected target” concept is meaningful only at the instant that an observation process—in the form of the standard multitarget likelihood function *f_k_*{*Z_k_*|*X*)—is applied.
2. Section 5.2: A more useful formula for *f_k_*{*Z_k_*|*X*), Equation (57).
3. Section 5.3: Thedetected-target likelihood function $f_{k}^{d}(Z_{k},Y|X)$, Equation (69).
4. Section 5.4: The detected-target density $f_{k|k}^{d}(Y|Z_{1:k})$, Equation (75).
5. Section 5.5: The undetected-target likelihood function $f_{k}^{u}(Z_{k},V|X)$, Equation (83).
6. Section 5.6: The undetected-target density $f_{k|k}^{u}(V|Z_{1:k})$, Equation (90).
7. Section 5.7: The measurement-updated PGFL and its associated detected-target and undetected-target PGFLs, Equations (97), (100), and (101).
8. Section 5.8: Analysis of the “undetected target” interpretation.
9. Section 5.9: The detected-target and undetected-target PGFLs when the prior PGFL is Bernoulli, Equations (110) and (112).

### 5.1. The “Undetected Target” Concept

At its most elemental level, the concept of “detected” vs. “undetected” target at time *t_k_* is independent of previous measurement history. The multitarget predicted distribution *f_k_*_:*k*-1_(*X*|*Z*_1:*k*-1_) determines how probable any given multitarget state-set *X* will be at time *t_k_*. However, only the current multitarget likelihood function *f_k_*(*Z*|*X*) determines which elements of *X* are detected vs. undetected at time *t_k_*.

The question then becomes: Given a finite subset *X* ⊆ ℑ, which elements of *X* generated measurements in *Z_k_* and which did not? The most that we can say is that, for each *Y* ⊆ *X*, there is some probability $p_{k}^{d}(Y|X)$ that all elements of *Y* ⊆ *X* generated measurements in *Z_k_*. The detected-target set is, therefore, a discrete RFS $\Xi_{k}^{d}$ ⊆ *X*. Likewise, there is some probability $p_{k}^{u}(V|X)$ that no elements of *V* ⊆ *X* generated measurements in *Z_k_*. The undetected-target set is therefore, a discrete RFS $\Xi_{k}^{u}$ ⊆ *X*with $\Xi_{k}^{u}=X-\Xi_{k}^{d}$. Given this, the following questions will be addressed:

1. How do we extend the discrete distributions $p_{k}^{d}(Y|X)$ and $p_{k}^{u}(V|X)$ to posterior probability densities $f_{k|k}^{d}(Y|Z_{1:k})$ and $f_{k|k}^{u}(V|Z_{1:k})$?
2. What are the PGFLs $G_{k|k}^{d}[h|Z_{1:k}]$ and $G_{k|k}^{u}[h|Z_{1:k}]$ of $f_{k|k}^{d}(Y|Z_{1:k})$ and $f_{k|k}^{u}(V|Z_{1:k})$?
3. If *G_k_*_|*k*−1_[*h*|*Z*_1:*k*−1_] has a given algebraic form, then what forms do $G_{k|k}^{d}[h|Z_{1:k}]$ and $G_{k|k}^{u}[h|Z_{1:k}]$ have?

### 5.2. The “Standard” Multitarget Likelihood Function

This is, for *Z_k_* = {**z**_1_, …, **z***_m_*} with |*Z*| = *m* and *X* = {**x**_1_, …, **x***_n_*} with |*X*| = *n*, [14] (Equation (12.139)), [13] (Equation (7.21)):

(54) $$L_{Z_{k}}(X)=f_{k}(Z_{k}|X)=e^{-\lambda_{k}}\kappa_{k}^{Z_{k}}{\left(1-p_{D}^{k}\right)}^{X}\sum_{\alpha \in A_{k}}\prod_{i:\alpha (i)>0}\begin{array}{cccc} \frac{p_{D}^{k}(x_{i})\cdot L_{z_{\alpha (i)}}(x_{i})}{\left(1-p_{D}^{k}(x_{i}))\cdot \kappa_{k}(z_{\alpha (i)}\right)} & & & \end{array}$$

where *α*:{1, …, *n*}→{0,1, …, *m*} is a measurement-to-track association (MTA) and *A_k_* is the set of all such associations at time *t_k_*. That is, *α* is such that *α*(*i*) = *α*(*i*′) > 0 implies *i* = *i*′. As usual, *κ_k_*(**z**) denotes the intensity function of the Poisson clutter process, *λ_k_*= ∫κ*_k_*(**z**)*d***z**, *L***_z_**(**x**) = *f_k_*(**z**|**x**) is the single-target likelihood function, and $p_{D}^{k}(x)$ is the state-dependent probability of detection. We will abbreviate *κ_k_*(**z**) = *κ*(**z**), *λ_k_*= *λ*, $p_{D}^{k}(x)=p_{D}(x)$, and *f_k_*(**z**|**x**) = *f*(**z**|**x**).

**Remark** **5.** *In general a multitarget state-set X will be labeled. However, the labeled version of Equation (54) is almost identical in form to Equation (54): f_k_*(Z|X) = *e^−λ^κ^Z^ if X is not labeled (not just if X =*∅*). Additionally, the “undetected target” concept was originally raised in the context of unlabeled RFSs. Thus, it is sufficient to use Equation (54).*

Choose a particular *α* ∈ *A*. Then *X^d^*^:^*^α^* = {**x***_i_*∈ *X*|*α*(*i*) > 0} is the set of **x***_i_*∈ *X* that—according to the hypothesis *α*—generated measurements in *Z*. Likewise, *X^u^*^:^*^α^* = {**x***_i_* ∈ *X*|*α*(*i*) = 0} is the set of those that did not. Now note the following:

MTAs are in one-to-one correspondence with pairs (*Y*,*τ*) where *Y* ⊆ *X* with |*Y*| ≤ |*Z_k_*| and where *τ*:*Y* ⇒ *Z_k_* is a one-to-one function (i.e., *τ*(**y**) = *τ*(**y**′) implies **y** = **y**′).

For on the one hand, let us be given *α*. Then define the pair (*Y_α_*,*τ_α_*) where *Y_α_*= {**x***_i_*∈ *X*|*α*(*i*) > 0} and *τ_α_*(**x***_i_*) = **z***_α_*_(*i*)_ for **x***_i_*∈ *Y*. On the other, let us be given a pair (*Y*,*τ*). Then for each *i* ∈ {1, …, *n*} define *α*_(*Y*,*τ*)_(*i*) = *j* if **x***_i_*∈ *Y* and *τ*(**x***_i_*) = z*_j_*; but *α*_(*Y*,*τ*)_(*i*) = 0 if otherwise—i.e., if there is no *j* ∈ {1, …, *m*} such that *τ*(x*_i_*) = **z***_j_*. It is easily verified that the transformations *α* ↦ (*Y*_α_,*τ_α_*) and (*Y*,*τ*) ↦ *α*_(*Y*,*τ*)_ are inverses of each other. 

Now define:

(55) $$L_{Z}^{*}(X)=f_{k}^{*}(Z|X)=\{\begin{array}{ccc} e^{-\lambda }\kappa^{Z}\sum_{\tau :Y\Rightarrow Z}\rho_{\tau }^{Y} & if & Y\neq \emptyset \\ e^{-\lambda }\kappa^{Z} & if & Y=\emptyset \end{array}$$

where the unitless ratio:

(56) $$\rho_{\tau }(y)=\frac{L_{\tau (y)}(y)}{\kappa (\tau (y))}$$

is a measure of how “target-like” vs. “clutter-like” the measurement *τ*(**y**) is (additionally, note that the “∗” in “$L_{Z}^{*}$” and “$f_{k}^{*}$” no longer refers to the label “ℓ*” in Section 4.4.)

Given this, note that the multitarget likelihood function can be rewritten as:

(57) $$L_{Z}(X)=e^{-\lambda }\kappa^{Z}\sum_{Y\subseteq X}\sum_{\tau :Y\Rightarrow Z}\begin{array}{ccc} {\left(1-p_{D}\right)}^{X-Y}p_{D}^{Y}\prod_{y\in Y}\frac{L_{\tau (y)}(y)}{\kappa (\tau (y))}=\sum_{Y\subseteq X}{\left(1-p_{D}\right)}^{X-Y}p_{D}^{Y}\cdot f_{k}^{*}(Z|Y). & & \end{array}$$

If *p_D_* = 1 then

(58) $$f_{Z}(X)=\sum_{Y\subseteq X}0^{X-Y}\cdot f_{k}^{*}(Z|Y)=f_{k}^{*}(Z|X).$$

It therefore, follows that $\int f_{k}^{*}(Z|X)\delta Z=1$ for all *X*. Thus,$f_{k}^{*}(Z|X)$ is the same thing as $f_{k}(Z|X)$, but under perfect-detection conditions.

For future reference note that if *Y* = {**y**_1_, …, **y***_n_*} with |*Y*| = *n*, then:

(59) $$\sum_{\tau :Y\Rightarrow Z}\rho_{\tau }^{Y}=\sum_{\begin{array}{l} (z_{1},\ldots ,z_{n})\in Z^{n}:\\ |\{z_{1},\ldots ,z_{n}\}|=n \end{array}}\frac{L_{z_{1}}(y_{1})}{\kappa (z_{1})}\cdots \frac{L_{z_{n}}(y_{n})}{\kappa (z_{n})}=n!\sum_{\left\{z_{1},\ldots ,z_{n}\}\in F_{n}(Z\right)}\begin{array}{ccc} \frac{L_{z_{1}}(y_{1})}{\kappa (z_{1})}\cdots \frac{L_{z_{n}}(y_{n})}{\kappa (z_{n})} & & \end{array}$$

where the third summation is taken over all {**z**_1_, …, **z***_n_*} ⊆ *Z* of cardinality *n* = |*Y*| ≤ |*Z*|.

### 5.3. The General Detected-Target Likelihood Function

Given these preliminaries, let *X* be a fixed finite subset of ℑ and define:

(60) $$p_{k}^{d}(Z_{k},Y|X)=1_{X}^{Y}\cdot {\left(1-p_{D}\right)}^{X-Y}p_{D}^{Y}\cdot f_{k}^{*}(Z_{k}|Y)$$

where, note, (**1***_X_*)*^Y^* = 1 if *Y* ⊆ *X* and (**1***_X_*)*^Y^*= 0 otherwise. This is a continuous density in *Z* and a discrete distribution in *Y*:

(61) $$\int p_{k}^{d}(Z,Y|X)\delta Z=1_{X}^{Y}\cdot {\left(1-p_{D}\right)}^{X-Y}p_{D}^{Y}$$

(62) $$\sum_{Y\subseteq X}\int p_{k}^{d}(Z,Y|X)\delta Z=\sum_{Y\subseteq X}{\left(1-p_{D}\right)}^{X-Y}p_{D}^{Y}={\left(1-p_{D}+p_{D}\right)}^{X}=1$$

where the final equation follows from Equation (11). Equation (61) is the probability that all of the elements of the subset *Y* of *X* generated measurements; and is largest when *p_D_*(**x**) ≈ 1 for **x** ∈ *Y* and *p_D_*(**x**) ≈ 0 for **x** ∈ *X* − *Y*, where “≈” denotes approximate equality. It is the distribution of the *detected-target RFS* in *X*:

(63) $$\mathrm{Pr}(\Xi_{k}^{d}=Y|X)=1_{X}^{Y}\cdot {\left(1-p_{D}\right)}^{X-Y}p_{D}^{Y}.$$

Equation (57) has the following interpretation: *L_Z_*(*X*) is the unweighted average of hypotheses $p_{k}^{d}(Z,Y|X)$ regarding the likelihood that subset *Y* of *X* generated measurements in *Z*. The factor ${\left(1-p_{D}\right)}^{X-Y}p_{D}^{Y}$ quantifies the “raw detectability” of *Y*, whereas $f_{k}^{*}(Z_{k}|Y)$ measures the degree to which detectability is degraded by clutter under perfect-detectability conditions.

We need to transform $p_{k}^{d}(Z,Y|X)$ so that it becomes a continuous density $f_{k}^{d}(Z,Y|X)$ with respect to *Y*. This is accomplished as follows. For *X* = {**x**_1_, …, **x***_n_*} with |*X*| = *n* and *Y* = {**y**_1_, …, **y***_ν_*} with |*Y*|=*ν*, define:

(64) $${\tilde{\delta }}_{X}(Y)=\{\begin{array}{ccc} 1 & if & Y=\emptyset \\ \sum_{\tau :\{1:\nu \}\Rightarrow \{1:n\}}\prod_{i=1}^{\nu }\delta_{x_{\tau (i)}}(y_{i}) & if & 0\leq \nu \leq n\\ 0 & if & \nu >n \end{array}$$

where the summation is taken over all one-to-one functions *τ*:{1, …, *ν*} ⇒ {1, …, *n*}. This is a multitarget density function with respect to Y.

Note that Equation (64) can be rewritten in the same form as Equation (59):

(65) $${\tilde{\delta }}_{X}(Y)=\sum_{\tau :Y\Rightarrow X}{\tilde{\rho }}_{\tau }=\nu !\sum_{\left\{x_{1},\ldots ,x_{\nu }\}\in F_{\nu }(X\right)}\begin{array}{ccc} \delta_{x_{1}}(y_{1})\cdots \delta_{x_{\nu }}(y_{\nu }) & & \end{array}$$

where the first summation is taken over all one-to-one functions *τ*:*Y* ⇒ *X* and where we define ${\tilde{\rho }}_{\tau }(y)=\delta_{\tau (y)}(y)$. Given this, in Section 6.1, Section 6.2 and Section 6.3 it is respectively shown that:

(66) $$\int {\tilde{\delta }}_{X}(Y)\delta Y=2^{\left|X\right|}$$

(67) $$\int {\tilde{\delta }}_{X}(Y)\cdot f(X)\delta X=\int f(Y\cup W)\delta W$$

(68) $$\int {\tilde{\delta }}_{X}(Y)\cdot {\left(1-p_{D}\right)}^{X-Y}p_{D}^{Y}\delta Y=\begin{array}{cc} 1 & \end{array}$$

where Equations (66)–(68) are true for all finite *X,Y* ⊆ ℑ and all multitarget densities *f*(*X*).

We are now in a position to define the *general detected-target likelihood function*:

(69) $$f_{k}^{d}(Z_{k},Y|X)={\tilde{\delta }}_{X}(Y)\cdot {\left(1-p_{D}\right)}^{X-Y}p_{D}^{Y}\cdot f_{k}^{*}(Z_{k}|Y).$$

It is the likelihood that, given a target-set *X*, the following are simultaneously true: *Z_k_* is the measurement-set collected at time *t_k_*; and *Y* ⊆ *X* is a subset of targets in *X* that generated measurements in *Z_k_*. In Section 6.4 the following is verified:

(70) $$\int f_{k}^{d}(Z_{k},Y|X)\delta Y=f_{k}(Z_{k}|X)=L_{Z_{k}}(X).$$

### 5.4. The General Detected-Target Density

Let us be given the prior distribution *f_k_*_|*k*−1_(*X*|*Z*_1:*k*−1_). Since $f_{k}^{d}(Z_{k},Y|X)$ does not depend on *Z*_1:*k*−1_, then $f_{k}^{d}(Z_{k},Y|X)=f_{k}^{d}(Z_{k},Y|X,Z_{1:k-1})$ and so from Bayes’ rule and the total probability theorem we obtain:

(71) $$f_{k}^{d}(Z_{k},Y|Z_{1:k-1})=\int f_{k}^{d}(Z_{k},Y|X)\cdot f_{k|k-1}(X|Z_{1:k-1})\delta X.$$

This is the probability (density) that, at time *t_k_*, the measurement-set *Z_k_* will be collected, and that the elements of *Y* ⊆ ℑ generated measurements in *Z_k_*. 

Additionally, from Bayes’ rule, we get the general detected-target posterior density—i.e., the probability (density) that all of the elements of *Y* ⊆ ℑ generated measurements in *Z_k_*:

(72) $$f_{k}^{d}(Y|Z_{1:k})=\frac{f_{k}^{d}(Z_{k},Y|Z_{1:k-1})}{f_{k|k-1}(Z_{k}|Z_{1:k-1})}=\frac{f_{k}^{d}(Z_{k},Y|Z_{1:k-1})}{\int f_{k}^{d}(Z_{k},W|Z_{1:k-1})\delta W}.$$

It is the distribution of the *general detected-target RFS*$\Xi_{k|k}^{d}$ at time *t_k_*.

We thereby end up with the following specific formulas:

(73) $$f_{k}^{d}(Z_{k},Y|Z_{1:k-1})=f_{k}^{*}(Z_{k}|Y)\cdot p_{D}^{Y}\cdot \frac{\delta G_{k|k-1}}{\delta Y}[1-p_{D}]$$

(74) $$f_{k}(Z_{k}|Z_{1:k-1})=\int f_{k}^{*}(Z_{k}|Y)\cdot p_{D}^{Y}\cdot \frac{\delta G_{k|k-1}}{\delta Y}[1-p_{D}]\begin{array}{cc} \delta Y & \end{array}$$

(75) $$f_{k}^{d}(Y|Z_{1:k})=\frac{f_{k}^{*}(Z_{k}|Y)\cdot p_{D}^{Y}\cdot \frac{\delta G_{k|k-1}}{\delta Y}[1-p_{D}]}{\int f_{k}^{*}(Z_{k}|W)\cdot p_{D}^{W}\cdot \frac{\delta G_{k|k-1}}{\delta W}[1-p_{D}]\delta W}.$$

For, substituting Equation (69) for $f_{k}^{d}(Z_{k},Y|X)$ and applying Equation (67):

(76) $$f_{k}^{d}(Z_{k},Y|X)=\int {\tilde{\delta }}_{X}(Y)\cdot {\left(1-p_{D}\right)}^{X-Y}p_{D}^{Y}\cdot f_{k}^{*}(Z_{k}|Y)\cdot f_{k|k-1}(X|Z_{1:k-1})\delta X$$

(77) $$=p_{D}^{Y}\cdot f_{k}^{*}(Z_{k}|Y)\int {\tilde{\delta }}_{X}(Y)\cdot {\left(1-p_{D}\right)}^{X-Y}\cdot f_{k|k-1}(X|Z_{1:k-1})\delta X$$

(78) $$=p_{D}^{Y}\cdot f_{k}^{*}(Z_{k}|Y)\int {\left(1-p_{D}\right)}^{(Y\cup V)-Y}\cdot f_{k|k-1}(Y\cup V|Z_{1:k-1})\delta V$$

(79) $$=p_{D}^{Y}\cdot f_{k}^{*}(Z_{k}|Y)\int {\left(1-p_{D}\right)}^{V}\cdot f_{k|k-1}(Y\cup V|Z_{1:k-1})\delta V$$

(80) $$=p_{D}^{Y}\cdot f_{k}^{*}(Z_{k}|Y)\cdot \frac{\delta G_{k|k-1}}{\delta Y}[1-p_{D}]$$

where the final equation follows from Equation (11.251) of [14].

### 5.5. The General Undetected-Target Likelihood Function

The detected-target RFS $\Xi_{k}^{d}\subseteq X$ was defined in Equation (63). By definition, the undetected-target RFS in *X* is $\Xi_{k}^{u}=X-\Xi_{k}^{d}$. For all *V* ⊆ *X*, note that:

(81) $$\mathrm{Pr}(\Xi_{k}^{u}V|X)=\mathrm{Pr}(\Xi_{k}^{d}X-V|X)=1_{X}^{V}\cdot {\left(1-p_{D}\right)}^{V}p_{D}^{X-V}.$$

(Note that (**1***_X_*)*^V^* should be used rather than (**1***_X_*)*^X-V^*—the latter is incorrect because it does not force *V* to be a subset of *X*. For example, let *V* = {**u**} where **x** ∉ *X*. Then it should be the case that $\mathrm{Pr}(\Xi_{k}^{u}\{x\}|X)=0$. However, if we instead use (**1***_X_*)*^X-V^*, then since (**1***_X_*)*^X-^*^{**x**}^ = (**1***_X_*)*^X^* = 1 we would get the incorrect result $\mathrm{Pr}(\Xi_{k}^{u}\{x\}|X)\neq 0$.)

Additionally, note that $\mathrm{Pr}(\Xi_{k}^{u}V|X)$ is largest when *p_D_*(**x**) ≈ 0 if **x** ∈ *V* and *p_D_*(**x**) ≈ 1 if **x**∈ *X* − *V*—in which case all of the elements of *X* − *V* should generate measurements in the manner described by $f_{k}^{*}(Z_{k}|X-V)$. The undetected-target analog of Equation (60) is, therefore:

(82) $$p_{k}^{u}(Z_{k},V|X)=1_{X}^{V}\cdot {\left(1-p_{D}\right)}^{V}p_{D}^{X-V}\cdot f_{k}^{*}(Z_{k}|X-V).$$

From here on, the analysis for $\Xi_{k}^{u}$ proceeds in the same manner as that for $\Xi_{k}^{d}$. That is, replace $p_{k}^{u}(Z_{k},V|X)$ with:

(83) $$f_{k}^{u}(Z_{k},V|X)={\tilde{\delta }}_{X}(V)\cdot {\left(1-p_{D}\right)}^{V}p_{D}^{X-V}\cdot f_{k}^{*}(Z_{k}|X-V).$$

This is the *general undetected-target likelihood function*—i.e., the likelihood that, given a target-set *X*, the following are true: *Z_k_* is the set of generated measurements at time *t_k_*; and *V* ⊆ *X* is a subset of targets in *X* that generated no measurements. Thus, by Equations (58) and (68):

(84) $$\int f_{k}^{u}(Z,V|X)\delta Z={\tilde{\delta }}_{X}(V)\cdot {\left(1-p_{D}\right)}^{V}p_{D}^{X-V}$$

(85) $$\int f_{k}^{u}(Z,V|X)\delta Z\delta V=\int {\tilde{\delta }}_{X}(V)\cdot {\left(1-p_{D}\right)}^{V}p_{D}^{X-V}\delta V=1.$$

### 5.6. The General Undetected-Target Density

Let *f_k_*_|*k*−1_(*X*|*Z*_1:*k*−1_) be the predicted multitarget distribution at time *t_k_*. Since $f_{k}^{u}(Z_{k},V|X)=f_{k}^{u}(Z_{k},V|X,Z_{1:k-1})$, from Bayes’ rule and the total probability theorem we obtain:

(86) $$f_{k}^{u}(Z_{k},V|Z_{1:k-1})=\int f_{k}^{u}(Z_{k},V|X)\cdot f_{k|k-1}(X|Z_{1:k-1})\delta X.$$

This is the probability (density) that, at time *t_k_*, the measurement-set *Z_k_* will be collected; and that none of the elements of *V* ⊆ ℑ generated measurements in *Z_k_*. Thus, the general undetected-target density—i.e., the probability (density) that none of the elements of *V* ⊆ ℑ generated measurements in *Z_k_*—is:

(87) $$f_{k}^{u}(V|Z_{1:k})=\frac{f_{k}^{u}(Z_{k},V|Z_{1:k-1})}{f_{k}(Z_{k}|Z_{1:k-1})}=\frac{f_{k}^{u}(Z_{k},V|Z_{1:k-1})}{\int f_{k}^{u}(Z_{k},W|Z_{1:k-1})\delta W}.$$

This leads to the following specific formulas:

(88) $$f_{k}^{u}(Z_{k},V|Z_{1:k-1})={\left(1-p_{D}\right)}^{V}\int p_{D}^{U}\cdot f_{k}^{*}(Z_{k}|U)\cdot f_{k|k-1}(V\cup U|Z_{1:k-1})\delta U$$

(89) $$f_{k}(Z_{k}|Z_{1:k-1})=\int f_{k}^{*}(Z_{k}|U)\cdot p_{D}^{U}\cdot \frac{\delta G_{k|k-1}}{\delta U}[1-p_{D}]\begin{array}{cc} \delta U & \end{array}$$

(90) $$f_{k}^{u}(V|Z_{1:k})=\frac{{\left(1-p_{D}\right)}^{V}\int f_{k}^{*}(Z_{k}|U)\cdot p_{D}^{U}\cdot f_{k|k-1}(V\cup U|Z_{1:k-1})\delta U}{\int f_{k}^{*}(Z_{k}|W)\cdot p_{D}^{W}\cdot \frac{\delta G_{k|k-1}}{\delta W}[1-p_{D}]\delta W}.$$

For, using Equation (67):

(91) $$f_{k}^{u}(Z_{k},V|Z_{1:k-1})=\int {\tilde{\delta }}_{X}(V)\cdot {\left(1-p_{D}\right)}^{V}p_{D}^{X-V}\cdot f_{k}^{*}(Z_{k}|X-V)\cdot f_{k|k-1}(X|Z_{1:k-1})\delta X$$

(92) $$=\int {\left(1-p_{D}\right)}^{V}p_{D}^{(V\cup U)-V}\cdot f_{k}^{*}(Z_{k}|(V\cup U)-V)\cdot f_{k|k-1}(V\cup U|Z_{1:k-1})\delta U$$

(93) $$={\left(1-p_{D}\right)}^{V}\int p_{D}^{U}\cdot f_{k}^{*}(Z_{k}|U)\cdot f_{k|k-1}(V\cup U|Z_{1:k-1})\delta U$$

and:

(94) $$f_{k}(Z_{k}|Z_{1:k-1})=\int {\left(1-p_{D}\right)}^{V}\int p_{D}^{U}\cdot f_{k}^{*}(Z_{k}|U)\cdot f_{k|k-1}(V\cup U|Z_{1:k-1})\delta U\delta V$$

(95) $$=\int p_{D}^{U}\cdot f_{k}^{*}(Z_{k}|U)\int {\left(1-p_{D}\right)}^{V}\cdot f_{k|k-1}(V\cup U|Z_{1:k-1})\delta V\delta U$$

(96) $$=\int p_{D}^{U}\cdot f_{k}^{*}(Z_{k}|U)\cdot \frac{\delta G_{k|k-1}}{\delta U}[1-p_{D}]\delta U$$

where the final equation results from Equation (11.251) of [14].

### 5.7. PGFLs of the Detected/Undetected Target Densities

The PGFL corresponding to $f_{k|k}^{d}(Y|Z_{1:k-1})$ (Equation (75)) is:

(97) $$G_{k|k}^{d}[h|Z_{`1:k}]=\int h^{Y}\cdot f_{k|k}^{d}(Y|Z_{1:k})\delta Y=\frac{\int f_{k}^{*}(Z_{k}|Y)\cdot {\left(hp_{D}\right)}^{Y}\cdot \frac{\delta G_{k|k-1}}{\delta Y}[1-p_{D}]\delta Y}{\int f_{k}^{*}(Z_{k}|W)\cdot p_{D}^{W}\cdot \frac{\delta G_{k|k-1}}{\delta W}[1-p_{D}]\delta W}.$$

The PGFL corresponding to $f_{k|k}^{u}(Y|Z_{1:k-1})$(Equation (87)) is simpler than $f_{k|k}^{u}(Y|Z_{1:k-1})$:

(98) $$G_{k|k}^{u}[h|Z_{`1:k}]=\int h^{V}\cdot f_{k|k}^{u}(V|Z_{1:k})\delta V$$

(99) $$=\frac{\int p_{D}^{U}\cdot f_{k}^{*}(Z_{k}|U)\cdot \left(\int h^{V}\cdot {\left(1-p_{D}\right)}^{V}\cdot f_{j|k-1}(V\cup U|Z_{1:k-1})\delta V\right)\delta U}{\int f_{k}^{*}(Z_{k}|W)\cdot p_{D}^{W}\cdot \frac{\delta G_{k|k-1}}{\delta W}[1-p_{D}]\delta W}$$

(100) $$=\frac{\int f_{k}^{*}(Z_{k}|U)\cdot p_{D}^{U}\cdot \frac{\delta G_{k|k-1}}{\delta U}[h(1-p_{D})]\delta U}{\int f_{k}^{*}(Z_{k}|W)\cdot p_{D}^{W}\cdot \frac{\delta G_{k|k-1}}{\delta W}[1-p_{D}]\delta W}$$

where the final equation results from Equation (11.251) of [14].

Finally, in Section 6.5 it is shown that:

(101) $$G_{k|k}[h|Z_{1:k}]=\frac{\int f_{k}^{*}(Z_{k}|U)\cdot {\left(hp_{D}\right)}^{U}\cdot \frac{\delta G_{k|k-1}}{\delta U}[h(1-p_{D})]\delta U}{\int f_{k}^{*}(Z_{k}|W)\cdot p_{D}^{W}\cdot \frac{\delta G_{k|k-1}}{\delta W}[1-p_{D}]\delta W}.$$

Thus, posterior PGFL at time *t_k_* is an amalgam of the undetected-target and detected-target PGFLs. Note that if *p_D_* = 1 (all targets are perfectly detectable) then $G_{k|k}^{u}[h|Z_{1:k}]=1$ (there are no undetected targets) and $G_{k|k}[h|Z_{1:k}]=G_{k|k}^{d}[h|Z_{1:k}]$ (all targets are detected).

### 5.8. Analysis of the "Undetected Target" Interpretation

Let us now apply the preceding analysis to the “undetected-target” interpretation, in which:

1. “undetected targets” are those that “…have never been detected…” [11] (p. 1103); and
2. “…detected targets cannot become undetected targets” [4] (p. 246).

In what follows it will be demonstrated that the second claim leads to a contradiction, whereas the first one appears to be consistent with the formal theory of undetected targets.

*Claim (2) Leads to a Contradiction*. According to Equation (100), $G_{k|k}^{u}[h|Z_{1:k}]$ is the PGFL of targets that are undetected only at time *t_k_*. According to Claim (2), if a target is undetected at *t_k_* then it was also undetected at times *t*_1_, …, *t_k−_*_1_. Given this, it must be the case that $G_{k|k}^{u}[h|Z_{1:k}]=F_{k|k}^{u}[h]=e^{D_{k|k}[h-1]}$—and, in particular, that $G_{k|k}^{u}[h|Z_{1:k}]$ is always Poisson. 

However, this is not true. For, examine the first steps of the U-PMBM filter. Begin with *G*_0|0_[*h*] = 1—i.e., no targets are initially present in the scene. Then $G_{1|0}[h]=e^{D_{B}^{1|0}[h-1]}$ where $D_{B}^{1|0}(x)$ is the PHD of the Poisson target-appearance RFS; and so $F_{1}[g,h]=e^{\kappa_{1}[g-1]+D_{B}^{1|0}[h(1+p_{D}^{1}L_{g-1}^{1})-1]}$ by Equation (20); and so *G*_1|1_[*h*|*Z*_1_] is a PMB PGFL as in Equation (36):

(102) $$G_{1|1}[h]=e^{D_{B}^{1|0}[(h-1)(1-p_{D}^{1})]}\prod_{z\in Z_{1}}\frac{\kappa_{1}(z)+D_{B}^{1|0}[hp_{D}^{1}L_{z}^{1}]}{\kappa_{1}(z)+D_{B}^{1|0}[p_{D}^{1}L_{z}^{1}]}.$$

In Section 6.6 and Section 6.7 it is respectively shown that:

(103) $$G_{1|1}^{u}[h]=e^{D_{B}^{1|0}[(h-1)(1-p_{D}^{1})]}$$

(104) $$G_{1|1}^{d}[h]=\prod_{z\in Z_{1}}\frac{\kappa_{1}(z)+D_{B}^{1|0}[hp_{D}^{1}L_{z}^{1}]}{\kappa_{1}(z)+D_{B}^{1|0}[p_{D}^{1}L_{z}^{1}]}.$$

These equations are consistent with the “undetected targets” interpretation, Equation (45), since:

(105) $$G_{1|1}[h|Z_{1}]=G_{1|1}^{u}[h|Z_{1}]\cdot G_{1|1}^{d}[h|Z_{1}]=F_{1|1}^{u}[h]\cdot F_{1|1}^{d}[h_{1}]=G_{k|k}^{UP}[h].$$

From Equation (20), the next predicted PGFL is PMB:

(106) $$G_{1|1}^{u}[h]=e^{D_{B}^{2|1}[h-1]}\cdot G_{1|1}[1+p_{S}^{2|1}M_{h-1}^{2|1}|Z_{1}]$$

(107) $$=e^{D_{B}^{2|1}[h-1]+D_{B}^{1|0}[(1-p_{D}^{1})p_{S}^{2|1}M_{h-1}^{2|1}]}\prod_{z\in Z_{1}}\frac{\kappa_{1}(z)+D_{B}^{1|0}[(1+p_{S}^{2|1}M_{h-1}^{2|1})p_{D}^{1}L_{z}^{1}]}{\kappa_{1}(z)+D_{B}^{1|0}[p_{D}^{1}L_{z}^{1}]}.$$

It can be shown that $G_{2|2}^{u}[h|Z_{1:2}]$ is PMB, not Poisson. The claim that “detected targets” cannot become “undetected targets”, therefore, leads to a contradiction. The proof of this fact for general $G_{2|2}^{u}[h|Z_{1:2}]$ will not be proved here, since it suffices to address the following informative special case. Let $D_{B}^{2|1}=0$, $p_{D}^{1}=1$, and |*Z*_1_| = 1, so that *G*_2|1_[*h*|*Z*_1_] is Bernoulli. Then in Section 5.9 we will determine $G_{2|2}^{u}[h|Z_{1:2}]$ and $G_{2|2}^{d}[h|Z_{1:2}]$ and show that the former is Bernoulli—i.e., not Poisson.

*Claim (1) is Consistent with the Formal Theory of Undetected Targets*. Alter the preceding argument as follows. Instead of predicting *G*_1|1_[*h*|Z_1_], predict its Poisson factor $G_{1|1}^{u}[h|Z_{1}]=F_{1|1}^{u}[h]$ to obtain $G_{2|1}^{u}[h|Z_{1}]=e^{D_{B}^{1|0}[(1-p_{D}^{1})p_{S}^{2|1}M_{h-1}^{2|1}]}=F_{2|1}^{u}[h]$. Next, determine the undetected-target posterior PGFL of $F_{21|1}^{u}[h]$—i.e., the PGFL of those targets undetected at times *t*_1_,*t*_2_. According to Equation (105), since $F_{2|1}^{u}[h]$ is Poisson it is equal to $F_{2|2}^{u}[h]$. Then predict $F_{2|2}^{u}[h]$ to obtain the Poisson PGFL $F_{3|2}^{u}[h]$. Determine the undetected-target posterior PGFL of $F_{3|2}^{u}[h]$—i.e., the PGFL of those targets undetected at times *t*_1_,*t*_2_, *t*_3_. According to Equation (105), it is equal to $F_{3|3}^{u}[h]$. Repeat in this manner. At time *t_k_*, determine the unpredicted-target posterior PGFL of the Poisson PGFL $F_{k|k-1}^{u}[h]$. It is the PGFL of those targets undetected at times *t*_1_, …, *t_k_* and is equal to $F_{k|k}^{u}[h]$.

### 5.9. Undetected/Detected-Target PGFLs for a Bernoulli Prior

Suppose that *G_k_*_|*k*−1_[*h*|*Z*_1:*k*−1_] = 1 − *q* + *q*·*s*[*h*] is Bernoulli. Then the measurement-updated PGFL is Bernoulli [14] (p. 520):

*G_k_*_|*k*_[*h*] = 1 − *q*^+^+*q*^+^·*s*^+^[*h*] (108)

(109) $$q^{+}=\frac{q-qs[p_{D}]+q\sum_{z\in Z_{k}}\frac{s[p_{D}L_{z}]}{\kappa (z)}}{1-qs[p_{D}]+q\sum_{z\in Z_{k}}\frac{s[p_{D}L_{z}]}{\kappa (z)}},s^{+}(x)=\frac{q-qp_{D}(x)+q\sum_{z\in Z_{k}}\frac{p_{D}(x)\cdot L_{z}(x)}{\kappa (z)}}{1-qs[p_{D}]+q\sum_{z\in Z_{k}}\frac{s[p_{D}L_{z}]}{\kappa (z)}}\cdot s(x).$$

In Section 6.9 it is shown that the detected-target posterior PGFL is Bernoulli:

(110) $$G_{k|k}^{u}[h]=1-q^{d}+q^{d}\cdot s^{d}[h]$$

(111) $$q^{d}=\frac{q\sum_{z\in Z_{k}}\frac{s[p_{D}L_{z}]}{\kappa (z)}}{1-qs[p_{D}]+q\sum_{z\in Z_{k}}\frac{s[p_{D}L_{z}]}{\kappa (z)}},s^{d}(x)=\frac{\sum_{z\in Z_{k}}\frac{p_{D}(x)\cdot L_{z}(x)}{\kappa (z)}}{\sum_{z\in Z_{k}}\frac{s[p_{D}L_{z}]}{\kappa (z)}}\cdot s(x).$$

In Section 6.8 it is shown that the undetected-target posterior PGFL is not Poisson:

(112) $$G_{k|k}^{u}[h]=1-q^{u}+q^{u}\cdot s^{u}[h]$$

(113) $$q^{u}=\frac{q-qs[p_{D}]}{1-qs[p_{D}]+q\sum_{z\in Z_{k}}\frac{s[p_{D}L_{z}]}{\kappa (z)}},s^{u}(x)=\frac{1-p_{D}(x)}{s[1-p_{D}]}\cdot s(x).$$

Here, *q^u^* is the probability that the posterior undetected-target RFS is nonempty—i.e., it is the target’s *composite probability of undetectability*. Note that *q^u^*+ *q^d^*= *q^+^*.

Additionally, note that *q^u^* parses the distinction between nonexistent vs. undetectable targets. If *q*> 0 (the target exists) and *p_D_* = 0 (it is undetectable), then no information can be collected about it and so its composite undetectability is *q^u^*= *q*. For example, if *q* = 1 then *q^u^* = 1—i.e., if a definitely-existing target is undetectable then it is compositely undetectable. 

At the other extreme, if *q* = 0 (it does not exist) then *q^u^* = 0 (it is compositely detectable: 1 − *q^u^* = 1). This seems counter-intuitive since a nonexistent target would seem to be inherently undetectable. However, a nonexistent target is neither detectable nor undetectable. Whereas an existent target can generate either an actual measurement **z** or the null measurement ∅, a nonexistent target cannot generate any measurement. Thus, a nonexistent target has been “detected” if, as must be the case, it has not generated any measurement. In this sense, all nonexistent targets are compositely detectable. 

Now suppose that *q* = 1—i.e., that the target definitely exists. Then:

(114) $$q^{u}=\frac{s[1-p_{D}]}{s[1-p_{D}]+\sum_{z\in Z_{k}}\frac{s[p_{D}L_{z}]}{\kappa (z)}}.$$

That is, the target’s composite undetectability *q^u^* is a composite of its “raw undetectability” *s*[1 − *p_D_*] and the degree to which clutter density impairs its detectability. It varies between *q^u^* = 0 when *κ* = 0 and *q^u^* = 1 when *κ* = ∞. That is, the composite undetectability of a definitely-existing target is 0 if there is no clutter; and its composite detectability 1 − *q^u^* is 0 if the clutter density is infinite (and, thus, the signal-to-noise ratio is extremely small).

## 6. Mathematical Derivations

### 6.1. Proof of Equation (66)

Recall that *n*!*C*_|*X*|,*n*_ is the number permutations of the elements of *X* taken *n* at a time; and that *C*_|*X*|,*n*_ is the number of subsets of *X* of cardinality *n*. Then:

(115) $$\int {\tilde{\delta }}_{X}(Y)\delta Y=\begin{array}{cc} \sum_{n=0}^{\left|X\right|}\frac{1}{n!}\int \sum_{\tau :\{1:n\}\Rightarrow \{1:|X|\}} & \left(\prod_{i=1}^{n}\delta_{\tau (i)}(y_{i})\right) \end{array}dy_{1}\cdots dy_{n}$$

(116) $$=\begin{array}{cccc} \sum_{n=0}^{\left|X\right|}\frac{1}{n!}\sum_{\tau :\{1:n\}\Rightarrow \{1:|X|\}}1 & = & \sum_{n=0}^{\left|X\right|}\frac{n!}{n!}\cdot C_{|X|,n}=\sum_{n=0}^{\left|X\right|}C_{|X|,n}=2^{\left|X\right|}. & \end{array}$$

### 6.2. Proof of Equation (67)

Let *ν* = |*Y*| and recall from Equation (65) that:

(117) $$\sum_{\tau :\{1:\nu \}\Rightarrow \{1:n\}}\delta_{x_{\tau (1)}}(y_{1})\cdots \delta_{x_{\tau (\nu )}}(y_{\nu })=\nu !\sum_{\left\{x_{1},\ldots ,x_{\nu }\}\in F_{\nu }(\{x_{1},\ldots ,x_{n}\}\right)}\begin{array}{ccc} \delta_{x_{1}}(y_{1})\cdots \delta_{x_{\nu }}(y_{\nu }) & & \end{array}$$

where *F_ν_*(*X*) denotes the set of subsets of *X* of cardinality *ν*. Then:

(118) $$\int {\tilde{\delta }}_{X}(Y)\cdot f(X)\delta X=\sum_{n\geq \nu }\frac{\nu !}{n!}\int \sum_{\left\{w_{1},\ldots ,w_{\nu }\}\in F_{\nu }(\{x_{1},\ldots ,x_{n}\}\right)}\begin{array}{ccc} \delta_{w_{1}}(y_{1})\cdots \delta_{w_{\nu }}(y_{\nu })\cdot f(\{x_{1},\ldots ,x_{n}\})dx_{1}\cdots dx_{n} & & \end{array}$$

(119) $$=\sum_{n\geq \nu }\frac{\nu !}{n!}\int \sum_{\left\{w_{1},\ldots ,w_{\nu }\}\in F_{\nu }(\{x_{1},\ldots ,x_{n}\}\right)}\begin{array}{ccc} \delta_{w_{1}}(y_{1})\cdots \delta_{w_{\nu }}(y_{\nu })\cdot f(\{w_{1},\ldots ,w_{\nu },x_{1},\ldots ,x_{n-\nu }\})dw_{1}\cdots dw_{\nu }dx_{1}\cdots dx_{n-\nu } & & \end{array}$$

(120) $$=\sum_{n\geq \nu }\frac{\nu !}{n!}\int \left(\sum_{\left\{y_{1},\ldots ,y_{\nu }\}\in F_{\nu }(\{x_{1},\ldots ,x_{n}\}\right)}1\right)\begin{array}{ccc} \cdot f(\{y_{1},\ldots ,y_{\nu },x_{1},\ldots ,x_{n-\nu }\})dx_{1}\cdots dx_{n-\nu } & & \end{array}$$

(121) $$=\sum_{n\geq \nu }\frac{\nu !}{n!}\int C_{n,\nu }\begin{array}{ccc} \cdot f(\{y_{1},\ldots ,y_{\nu },x_{1},\ldots ,x_{n-\nu }\})dx_{1}\cdots dx_{n-\nu } & & \end{array}$$

(122) $$=\sum_{n\geq \nu }\frac{1}{(n-\nu )!}\int \begin{array}{ccc} f(\{y_{1},\ldots ,y_{\nu },x_{1},\ldots ,x_{n-\nu }\})dx_{1}\cdots dx_{n-\nu } & & \end{array}$$

(123) $$=\sum_{j\geq 0}\frac{1}{j!}\int \begin{array}{c} f(\{y_{1},\ldots ,y_{\nu },x_{1},\ldots ,x_{j}\})dx_{1}\cdots dx_{j}=\int f(\{y_{1},\ldots ,y_{\nu }\}\cup W)\delta W. \end{array}$$

### 6.3. Proof of Equation (68)

Note that:

(124) $$\int {\tilde{\delta }}_{X}(Y)\cdot {\left(1-p_{D}\right)}^{X-Y}p_{D}^{Y}\delta Y={\left(1-p_{D}\right)}^{X}\begin{array}{ccc} \int {\tilde{\delta }}_{X}(Y)\cdot {\left(\frac{p_{D}}{1-p_{D}}\right)}^{Y}\delta Y & & \end{array}$$

(125) $$={\left(1-p_{D}\right)}^{X}\begin{array}{ccc} \sum_{n=0}^{\left|X\right|}\frac{1}{n!}\int \sum_{\tau :\{1:n\}\Rightarrow \{1:|X|\}}\delta_{x_{\tau (1)}}(y_{1})\cdots \delta_{x_{\tau (n)}}(y_{n})\cdot \frac{p_{D}(y_{1})}{1-p_{D}(y_{1})}\cdots \frac{p_{D}(y_{n})}{1-p_{D}(y_{n})}dy_{1}\cdots dy_{n} & & \end{array}$$

(126) $$={\left(1-p_{D}\right)}^{X}\begin{array}{ccc} \sum_{n=0}^{\left|X\right|}\frac{1}{n!}\int \sum_{\tau :\{1:n\}\Rightarrow \{1:|X|\}}\frac{\delta_{x_{\tau (1)}}(y_{1})\cdot p_{D}(y_{1})}{1-p_{D}(y_{1})}\cdots \frac{\delta_{x_{\tau (n)}}(y_{n})\cdot p_{D}(y_{n})}{1-p_{D}(y_{n})}dy_{1}\cdots dy_{n} & & \end{array}$$

(127) $$={\left(1-p_{D}\right)}^{X}\begin{array}{ccc} \sum_{n=0}^{\left|X\right|}\frac{1}{n!}\sum_{\tau :\{1:n\}\Rightarrow \{1:|X|\}}\left(\int \frac{\delta_{x_{\tau (1)}}(y_{1})\cdot p_{D}(y_{1})}{1-p_{D}(y_{1})}dy_{1}\right)\cdots \left(\int \frac{\delta_{x_{\tau (n)}}(y_{n})\cdot p_{D}(y_{n})}{1-p_{D}(y_{n})}dy_{n}\right) & & \end{array}$$

(128) $$={\left(1-p_{D}\right)}^{X}\begin{array}{ccc} \sum_{n=0}^{\left|X\right|}\frac{1}{n!}\sum_{\tau :\{1:n\}\Rightarrow \{1:|X|\}}\frac{p_{D}(x_{\tau (1)})}{1-p_{D}(x_{\tau (1)})}\cdots \frac{p_{D}(x_{\tau (n)})}{1-p_{D}(x_{\tau (n)})} & & \end{array}$$

(129) $$={\left(1-p_{D}\right)}^{X}\begin{array}{ccc} \sum_{n=0}^{\left|X\right|}\frac{n!}{n!}\sum_{\left\{x_{1},\ldots ,x_{n}\}\in F_{n}(X\right)}\frac{p_{D}(x_{1})}{1-p_{D}(x_{1})}\cdots \frac{p_{D}(x_{n})}{1-p_{D}(x_{n})} & & \end{array}$$

(130) $$={\left(1-p_{D}\right)}^{X}\begin{array}{ccc} \sum_{n=0}^{\left|X\right|}\sum_{W\subseteq X,|W|=n}{\left(\frac{p_{D}}{1-p_{D}}\right)}^{W}={\left(1-p_{D}\right)}^{X}\sum_{W\subseteq X}{\left(\frac{p_{D}}{1-p_{D}}\right)}^{W} & & \end{array}$$

(131) $$=\begin{array}{c} {\left(1-p_{D}\right)}^{X}{\left(1+\frac{p_{D}}{1-p_{D}}\right)}^{X}=1 \end{array}$$

where the second-to-final equation follows from Equation (11).

### 6.4. Proof of Equation (70)

Begin by noting that:

(132) $$\int f_{k}^{d}(Z,Y|X)\delta Y=\int {\tilde{\delta }}_{X}(Y)\cdot {\left(1-p_{D}\right)}^{X-Y}p_{D}^{Y}\cdot f_{k}^{*}(Z_{k}|Y)\delta Y$$

(133) $$=e^{-\lambda }\kappa^{Z}{\left(1-p_{D}\right)}^{X}\int \left(\sum_{\tau \prime :Y\Rightarrow X}{\tilde{\rho }}_{\tau \prime }^{Y}\right){\left(\frac{p_{D}}{1-p_{D}}\right)}^{Y}\left(\sum_{\tau :Y\Rightarrow Z}\rho_{\tau }^{Y}\right)\delta Y$$

(134) $$=e^{-\lambda }\kappa^{Z}{\left(1-p_{D}\right)}^{X}\int \sum_{\tau :Y\Rightarrow Z}\sum_{\tau \prime :Y\Rightarrow X}{\left(\frac{{\tilde{\rho }}_{\tau \prime }p_{D}\rho_{\tau }}{1-p_{D}}\right)}^{Y}\delta Y$$

where ${\tilde{\rho }}_{\tau \prime }$ was defined in Equation (65). Apply Equations (59) and (65) to obtain:

(135) $$\frac{1}{e^{-\lambda }\kappa^{Z}}\int f_{k}^{d}(Z,Y|X)\delta Y={\left(1-p_{D}\right)}^{X}\int \sum_{n=0}^{\left|X\right|}\frac{1}{n!}\sum_{\tau :\{y_{1:n}\}\Rightarrow Z}\sum_{\tau \prime :\{y_{1:n}\}\Rightarrow X}\prod_{i=1}^{n}\frac{p_{D}(y_{i})\cdot \delta_{\tau \prime (y_{i})}(y_{i})\cdot L_{\tau (y_{i})}(y_{i})}{\kappa (\tau (y_{i}))\cdot (1-p_{D}(y_{i}))}dy_{1}\cdots dy_{n}$$

(136) $$={\left(1-p_{D}\right)}^{X}\int \sum_{n=0}^{\left|X\right|}\frac{{\left(n!\right)}^{2}}{n!}\sum_{\left\{z_{1},\ldots ,z_{n}\}\in F_{n}(Z\right)}\sum_{\left\{x_{1},\ldots ,x_{n}\}\in F_{n}(X\right)}\prod_{i=1}^{n}\frac{p_{D}(y_{i})\cdot \delta_{x_{i}}(y_{i})\cdot L_{z_{i}}(y_{i})}{\kappa (z_{i})\cdot (1-p_{D}(y_{i}))}dy_{1}\cdots dy_{n}$$

(137) $$={\left(1-p_{D}\right)}^{X}\sum_{n=0}^{\left|X\right|}n!\sum_{\left\{z_{1},\ldots ,z_{n}\}\in F_{n}(Z\right)}\sum_{\left\{x_{1},\ldots ,x_{n}\}\in F_{n}(X\right)}\prod_{i=1}^{n}\frac{p_{D}(x_{i})\cdot L_{z_{i}}(x_{i})}{\kappa (z_{i})\cdot (1-p_{D}(x_{i}))}$$

(138) $$={\left(1-p_{D}\right)}^{X}\sum_{n=0}^{\left|X\right|}\sum_{\left\{x_{1},\ldots ,x_{n}\}\in F_{n}(X\right)}\prod_{i=1}^{n}\frac{p_{D}(x_{i})}{1-p_{D}(x_{i})}\cdot n!\sum_{\left\{z_{1},\ldots ,z_{n}\}\in F_{n}(Z\right)}\prod_{i=1}^{n}\frac{L_{z_{i}}(x_{i})}{\kappa (z_{i})}$$

(139) $$={\left(1-p_{D}\right)}^{X}\sum_{n=0}^{\left|X\right|}\sum_{\left\{y_{1},\ldots ,y_{n}\}\in F_{n}(X\right)}\prod_{i=1}^{n}\frac{p_{D}(y_{i})}{1-p_{D}(y_{i})}\sum_{\tau :(y_{1:n})\Rightarrow Z_{k}}\prod_{i=1}^{n}\frac{L_{\tau (y_{i})}(y_{i})}{\kappa (\tau (y_{i}))}.$$

After applying Equation (59) again we obtain, as claimed,

(140) $$={\left(1-p_{D}\right)}^{X}\sum_{Y\subseteq X}{\left(\frac{p_{D}}{1-p_{D}}\right)}^{Y}\left(\sum_{\tau :Y\Rightarrow Z}\rho_{\tau }^{Y}\right)=\sum_{Y\subseteq X}{\left(1-p_{D}\right)}^{X-Y}p_{D}^{Y}\sum_{\tau :Y\Rightarrow Z}\rho_{\tau }^{Y}=\frac{1}{e^{-\lambda }\kappa^{Z}}\cdot f(Z|X).$$

### 6.5. Proof of Equation (101)

The PGFL of *f_k_*_|*k*_(*X*|*Z*_1:*k*_) is:

(141) $$G_{k|k}[h|Z_{1:k}]=\frac{\int h^{X}\cdot f_{k}(Z_{k}|X)\cdot f_{k|k-1}(X|Z_{1:k-1})\delta X}{\int f_{k}(Z_{k}|X)\cdot f_{k|k-1}(X|Z_{1:k-1})\delta X}.$$

Substituting Equation (69) for *f_k_*(*Z*|*X*) we obtain:

(142) $$G_{k|k}[h|Z_{1:k}]=\frac{\int h^{X}\cdot \left(\int {\tilde{\delta }}_{X}(Y)\cdot {\left(1-p_{D}\right)}^{X-Y}p_{D}^{Y}\cdot f_{k}^{*}(Z_{k}|Y)\delta Y\right)\cdot f_{k|k-1}(X|Z_{1:k-1})\delta X}{\int \left(\int {\tilde{\delta }}_{X}(Y)\cdot {\left(1-p_{D}\right)}^{X-Y}p_{D}^{Y}\cdot f_{k}^{*}(Z_{k}|Y)\delta Y\right)\cdot f_{k|k-1}(X|Z_{1:k-1})\delta X}$$

(143) $$=\frac{\int {\left(\frac{p_{D}}{1-p_{D}}\right)}^{Y}\cdot f_{k}^{*}(Z_{k}|Y)\cdot \left(\int {\tilde{\delta }}_{X}(Y)\cdot {\left(h(1-p_{D})\right)}^{X}\cdot f_{k|k-1}(X|Z_{1:k-1})\delta X\right)\delta Y}{\int {\left(\frac{p_{D}}{1-p_{D}}\right)}^{Y}\cdot f_{k}^{*}(Z_{k}|Y)\cdot \left(\int {\tilde{\delta }}_{X}(Y)\cdot {\left(1-p_{D}\right)}^{X}\cdot f_{k|k-1}(X|Z_{1:k-1})\delta X\right)\delta Y}$$

where, because of Equation (67):

(144) $$\int {\tilde{\delta }}_{X}(Y)\cdot {\left(h(1-p_{D})\right)}^{X}\cdot f_{k|k-1}(X|Z_{1:k-1})\delta X=\int {\left(h(1-p_{D})\right)}^{Y\cup U}\cdot f_{k|k-1}(Y\cup U|Z_{1:k-1})\delta U$$

(145) $$={\left(h(1-p_{D})\right)}^{Y}\int {\left(h(1-p_{D})\right)}^{U}\cdot f_{k|k-1}(Y\cup U|Z_{1:k-1})\delta U$$

(146) $$={\left(h(1-p_{D})\right)}^{Y}\cdot \frac{\delta G_{k|k-1}}{\delta Y}[h(1-p_{D})]$$

where the final equation follows from Equation (11.251) of [14].

Then, as claimed:

(147) $$G_{k|k}[h|Z_{1:k}]=\frac{\int {\left(\frac{p_{D}}{1-p_{D}}\right)}^{Y}\cdot f_{k}^{*}(Z_{k}|Y)\cdot {\left(h(1-p_{D})\right)}^{Y}\cdot \frac{\delta G_{k|k-1}}{\delta Y}[h(1-p_{D})]\delta Y}{\int {\left(\frac{p_{D}}{1-p_{D}}\right)}^{Y}\cdot f_{k}^{*}(Z_{k}|Y)\cdot {\left(1-p_{D}\right)}^{Y}\cdot \frac{\delta G_{k|k-1}}{\delta Y}[h(1-p_{D})]\delta Y}$$

(148) $$=\frac{\int f_{k}^{*}(Z_{k}|Y)\cdot {\left(hp_{D}\right)}^{Y}\cdot \frac{\delta G_{k|k-1}}{\delta Y}[h(1-p_{D})]\delta Y}{\int f_{k}^{*}(Z_{k}|Y)\cdot p_{D}^{}\cdot \frac{\delta G_{k|k-1}}{\delta Y}[h(1-p_{D})]\delta Y}.$$

### 6.6. Proof of Equation (103)

Begin by noting that:

(149) $$\frac{\delta G_{1|0}}{\delta Y}[h(1-p_{D})]=e^{D[h(1-p_{D})]}\cdot D^{Y}.$$

Then, from Equation (100) the undetected-target PGFL is, as claimed:

(150) $$G_{1|1}^{u}[h|Z_{1}]=\frac{\int f_{1}^{*}(Z_{`}{}_{1}|U)\cdot p_{D}^{U}\cdot \frac{\delta G_{1|0}}{\delta U}[h(1-p_{D})]\delta U}{\int f_{1}^{*}(Z_{`}{}_{1}|U)\cdot p_{D}^{U}\cdot \frac{\delta G_{1|0}}{\delta U}[1-p_{D}]\delta U}=\frac{\int f_{1}^{*}(Z_{`}{}_{1}|U)\cdot p_{D}^{U}\cdot e^{D[h(1-p_{D})]}\cdot D^{U}\delta U}{\int f_{1}^{*}(Z_{`}{}_{1}|U)\cdot p_{D}^{U}\cdot e^{D[1-p_{D}]}\cdot D^{U}\delta U}$$

(151) $$=\frac{e^{D[h(1-p_{D})]}\int f_{1}^{*}(Z_{`}{}_{1}|U)\cdot p_{D}^{U}\cdot D^{U}\delta U}{e^{D[1-p_{D}]}\int f_{1}^{*}(Z_{`}{}_{1}|U)\cdot p_{D}^{U}\cdot D^{U}\delta U}=e^{D[(h-1)(1-p_{D})]}.$$

### 6.7. Proof of Equation (104)

Begin by noting that:

(152) $$\frac{\delta G_{1|0}}{\delta Y}[1-p_{D}]=e^{-D[p_{D}]}\cdot D^{Y}.$$

From Equation (97):

(153) $$G_{1|1}^{d}[h|Z_{1}]=\frac{\int f_{1}^{*}(Z_{`}{}_{1}|U)\cdot {\left(hp_{D}\right)}^{U}\cdot \frac{\delta G_{1|0}}{\delta U}[1-p_{D}]\delta U}{\int f_{1}^{*}(Z_{`}{}_{1}|U)\cdot p_{D}^{U}\cdot \frac{\delta G_{1|0}}{\delta U}[1-p_{D}]\delta U}=\frac{\int f_{1}^{*}(Z_{`}{}_{1}|U)\cdot {\left(hp_{D}\right)}^{U}\cdot e^{-D[p_{D}]}\cdot D^{U}\delta U}{\int f_{1}^{*}(Z_{`}{}_{1}|U)\cdot p_{D}^{U}\cdot e^{-D[p_{D}]}\cdot D^{U}\delta U}$$

(154) $$=\frac{\int \left(\sum_{\tau :U\Rightarrow Z_{1}}\rho_{\tau }^{U}\right)\cdot {\left(hp_{D}D\right)}^{U}\delta U}{\int \left(\sum_{\tau :U\Rightarrow Z_{1}}\rho_{\tau }^{U}\right)\cdot {\left(p_{D}D\right)}^{U}\cdot \delta U}.$$

Abbreviate *D_h_* = *hp_D_D*. Then, from Equation (59):

(155) $$\int \left(\sum_{\tau :U\Rightarrow Z_{1}}\rho_{\tau }^{U}\right)\cdot {\left(hp_{D}D\right)}^{U}\delta U=\int \left(\sum_{\tau :U\Rightarrow Z_{1}}(\rho_{\tau }D_{h}{)}^{U}\right)\delta U$$

(156) $$=\sum_{n\geq 0}\frac{1}{n!}\int \sum_{(z_{1:n})\in Z_{1}^{n}:|\{z_{1:n}\}|=n}\frac{L_{z_{1}}(y_{1})\cdot D_{h}(y_{1})}{\kappa (z_{1})}\cdots \frac{L_{z_{1}}(y_{n})\cdot D_{h}(y_{n})}{\kappa (z_{n})}dy_{1}\cdots dy_{n}$$

(157) $$=\sum_{n\geq 0}\sum_{\left\{z_{1:n}\}\in F_{n}(Z_{1}\right)}\frac{D_{h}[L_{z_{1}}]}{\kappa (z_{1})}\cdots \frac{D_{h}[L_{z_{1}}]}{\kappa (z_{n})}=\sum_{n\geq 0}\sum_{W\subseteq Z_{1}:|Z_{1}|=n}\prod_{z\in W}\frac{D_{h}[L_{z}]}{\kappa (z)}$$

(158) $$=\sum_{W\subseteq Z_{1}}\prod_{z\in W}\frac{D_{h}[L_{z}]}{\kappa (z)}=\prod_{z\in Z_{1}}\left(1+\frac{D_{h}[L_{z}]}{\kappa (z)}\right)$$

where the final equation follows from Equation (11). Thus, as claimed:

(159) $$G_{1|1}^{d}[h|Z_{1}]=\frac{\prod_{z\in Z_{1}}\left(1+\frac{D_{h}[L_{z}]}{\kappa (z)}\right)}{\prod_{z\in Z_{1}}\left(1+\frac{D_{1}[L_{z}]}{\kappa (z)}\right)}=\prod_{z\in Z_{1}}\frac{\kappa (z)+D[hp_{D}L_{z}]}{\kappa (z)+D[p_{D}L_{z}]}.$$

### 6.8. Proof of Equation (113)

First note that:

(160) $$\frac{\delta G_{1|0}}{\delta Y}[h]=\{\begin{array}{ccc} 1-q+qs[h] & if & Y=\emptyset \\ qs(y) & if & Y=\{y\}\\ 0 & if & |Y|\geq 2 \end{array}.$$

From Equation (100), the numerator of the undetected-target PGFL is:

(161) $$\int f_{k}^{*}(Z_{`}{}_{k}|U)\cdot p_{D}^{U}\cdot \frac{\delta G_{k|k-1}}{\delta U}[h(1-p_{D})]\delta U$$

(162) $$=f_{k}^{*}(Z_{k}|\emptyset )\cdot G_{k|k-1}[h(1-p_{D})]+\int f_{k}^{*}(Z_{k}|\{y\})\cdot p_{D}(y)\cdot \frac{\delta G_{k|k-1}}{\delta y}[h(1-p_{D})]dy$$

(163) $$=e^{-\lambda }\kappa^{Z_{k}}\cdot (1-q+qs[h(1-p_{D})])+e^{-\lambda }\kappa^{Z_{k}}\int \sum_{z\in Z_{k}}\frac{L_{z}(y)}{\kappa (z)}\cdot p_{D}(y)\cdot qs(y)dy$$

(164) $$=e^{-\lambda }\kappa^{Z_{k}}\cdot (1-q+qs[h(1-p_{D})])+e^{-\lambda }\kappa^{Z_{k}}\sum_{z\in Z_{k}}\frac{qs[p_{D}L_{z}]}{\kappa (z)}.$$

Thus, as claimed, the undetected-target PGFL of a Bernoulli RFS is Bernoulli:

(165) $$G_{k|k}^{u}[h|Z_{1:k}]=\frac{1-q+qs[h(1-p_{D})]+\sum_{z\in Z_{k}}\frac{qs[p_{D}L_{z}]}{\kappa (z)}}{1-qs[p_{D}]+\sum_{z\in Z_{k}}\frac{qs[p_{D}L_{z}]}{\kappa (z)}}$$

(166) $$=\frac{1-q+qs[h(1-p_{D})]}{1-qs[p_{D}]+\sum_{z\in Z_{k}}\frac{qs[p_{D}L_{z}]}{\kappa (z)}}+\frac{qs[1-p_{D}]}{1-qs[p_{D}]+\sum_{z\in Z_{k}}\frac{qs[p_{D}L_{z}]}{\kappa (z)}}\cdot \frac{s[h(1-p_{D})]}{s[1-p_{D}]}$$

(167) $$=1-q^{u}+q^{u}s^{u}[h].$$

### 6.9. Proof of Equation (110)

Due to Equation (160), the numerator of the detected-target PGFL is:

(168) $$\int f_{k}^{*}(Z_{`}{}_{k}|U)\cdot {\left(hp_{D}\right)}^{U}\cdot \frac{\delta G_{k|k-1}}{\delta U}[1-p_{D}]\delta U$$

(169) $$=f_{k}^{*}(Z_{k}|\emptyset )\cdot G_{k|k-1}[1-p_{D}]+\int f_{k}^{*}(Z_{k}|\{y\})\cdot h(y)\cdot p_{D}(y)\cdot \frac{\delta G_{k|k-1}}{\delta y}[1-p_{D}]dy$$

(170) $$=e^{-\lambda }\kappa^{Z_{k}}\cdot (1-qs[p_{D}])+e^{-\lambda }\kappa^{Z_{k}}\int \sum_{z\in Z_{k}}\frac{L_{z}(y)}{\kappa (z)}\cdot h(y)\cdot p_{D}(y)\cdot qs(y)dy$$

(171) $$=e^{-\lambda }\kappa^{Z_{k}}\cdot (1-qs[p_{D}])+e^{-\lambda }\kappa^{Z_{k}}\sum_{z\in Z_{k}}\frac{qs[hp_{D}L_{z}]}{\kappa (z)}.$$

Thus, the detected-target PGFL is:

(172) $$G_{k|k}^{u}[h|Z_{1:k}]=\frac{1-qs[p_{D}]+\sum_{z\in Z_{k}}\frac{qs[hp_{D}L_{z}]}{\kappa (z)}}{1-qs[p_{D}]+\sum_{z\in Z_{k}}\frac{qs[p_{D}L_{z}]}{\kappa (z)}}=\frac{1-qs[p_{D}]}{1-qs[p_{D}]+\sum_{z\in Z_{k}}\frac{qs[p_{D}L_{z}]}{\kappa (z)}}+\frac{q\sum_{z\in Z_{k}}\frac{s[hp_{D}L_{z}]}{\kappa (z)}}{1-qs[p_{D}]+\sum_{z\in Z_{k}}\frac{qs[p_{D}L_{z}]}{\kappa (z)}}\cdot \frac{\sum_{z\in Z_{k}}\frac{s[hp_{D}L_{z}]}{\kappa (z)}}{\sum_{z\in Z_{k}}\frac{s[p_{D}L_{z}]}{\kappa (z)}}.$$

## 7. Conclusions

This review paper has assessed and compared the following proposed exact closed-form solutions of the multitarget Bayes filter:

1. Generalized labeled multi-Bernoulli (GLMB) filter [5,6,7,8].
2. Labeled multi-Bernoulli mixture (LMBM) filter [10].
3. Poisson multi-Bernoulli mixture (PMBM) filter, in three distinct versions:
  - “Unlabeled” or U-PMBM filter [11].
  - “Label-augmented” or LA-PMBM filter [12].
  - “Hybrid labeled-unlabeled” or H-PMBM filter [4].

It has been shown that:

1. The GLMB, LMBM filters solve the labeled multitarget Bayes filter in exact closed form.
2. They are, therefore, true Bayesian multitarget trackers.
3. The U-PMBM filter solves the unlabeled multitarget Bayes filter in exact closed form.
4. The “undetected-targets” interpretation of the U-PMBM filter appears to be valid.
5. The claim that detected targets cannot become undetected does not.
6. The equation $f_{k|k}(X|Z_{1:k})=f_{k|k}^{d}(X^{d}|Z_{1:k})\cdot f_{k|k}^{u}(X^{u}|Z_{1:k})$ in [4] is untrue.
7. It is theoretically impossible to prune U-PMBM distributions in a practical manner.
8. The U-PMBM, LA-PMBM, and H-PMBM filters are not true multitarget trackers.
9. The LA-PMBM and H-PMBM filters are theoretically and physically questionable.
10. In particular, the H-PMBM filter does not solve the “hybrid” multitarget Bayes filter in exact closed form.
